# Small extracellular vesicles from dental follicle stem cells provide biochemical cues for periodontal tissue regeneration

**DOI:** 10.1186/s13287-022-02767-6

**Published:** 2022-03-03

**Authors:** Liya Ma, Nanquan Rao, Hui Jiang, Yuzhe Dai, Songtao Yang, Hefeng Yang, Jiangtian Hu

**Affiliations:** 1 Yunnan Key Laboratory of Stomatology and Department of Dental Research, The Affiliated Stomatology Hospital of Kunming Medical University, Kunming, 650500 Yunnan People’s Republic of China; 2 Department of Orthodontics, The Affiliated Stomatology Hospital of Kunming Medical University, Kunming, 650500 Yunnan People’s Republic of China

**Keywords:** Small extracellular vesicles, Dental follicle stem cells, Periodontal ligament stem cells, Periodontal tissue regeneration

## Abstract

**Background:**

Treatments based on stem cell-derived small extracellular vesicles (sEVs) have been explored as an alternative to stem cell transplantation-based therapies in periodontal regeneration. Dental follicle stem cells (DFSCs) have shown great potential for regenerative medicine applications. However, it is unclear whether sEVs derived from DFSCs (DFSCs-sEVs) could be used in periodontal regeneration. This study investigates whether DFSCs-sEVs could regenerate damaged periodontal tissue and the potential underlying mechanism.

**Methods:**

DFSCs-sEVs were isolated and identified, and periodontal ligament stem cells (PDLSCs) were cocultured with the isolated sEVs. The effect of DFSCs-sEVs on the biological behaviour of PDLSCs was examined using EdU assay, CCK-8 assay, cell cycle analysis, wound healing, alizarin red staining, qRT-PCR, and western blot analysis. RNA sequencing and functional enrichment analysis were used to detect the signal pathway involved in the effect of DFSCs-sEVs on PDLSCs. PDLSCs were pretreated with ERK1/2 or p38 MAPK inhibitors to investigate the possible involvement of the ERK1/2 and p38 MAPK pathways. Additionally, DFSCs-sEVs were combined with collagen sponges and transplanted into the periodontal defects in SD rats, and then, pathological changes in periodontal tissue were examined using haematoxylin and eosin (HE) staining and micro-CT.

**Results:**

PDLSCs could internalize DFSCs-sEVs, thereby enhancing the proliferation assessed using EdU assay, CCK-8 assay and cell cycle analysis. DFSCs-sEVs significantly enhanced the migration of PDLSCs. DFSCs-sEVs promoted osteogenic differentiation of PDLSCs, showing deep Alizarin red staining, upregulated osteogenic genes (*RUNX2*, *BSP*, *COL1*), and upregulated protein expression (RUNX2, BSP, COL1, ALP). We found that p38 MAPK signalling was activated via phosphorylation. Inhibition of this signalling pathway with a specific inhibitor (SB202190) partially weakened the enhanced proliferation. After DFSCs-sEVs transplantation, new periodontal ligament-like structures and bone formation were observed in the damaged periodontal area in rats. Labelled DFSCs-sEVs were observed in the newly formed periodontal ligament and soft tissue of the defect area.

**Conclusions:**

Our study demonstrated that DFSCs-sEVs promoted periodontal tissue regeneration by promoting the proliferation, migration, and osteogenic differentiation of PDLSCs. The effect of DFSCs-sEVs in promoting PDLSCs proliferation may be partially attributed to the activation of p38 MAPK signalling pathway. DFSCs-sEVs provide us with a novel strategy for periodontal regeneration in the future.

**Supplementary Information:**

The online version contains supplementary material available at 10.1186/s13287-022-02767-6.

## Background

Periodontal disease is one of the most common human oral diseases in adults; it is the main cause of adult tooth loss and is characterized by the irreversible loss of connective tissue and bone tissue. The most ideal treatment for periodontal disease is to regenerate periodontal tissue, that is, to insert functional periodontal ligament comprising collagenous bundles between the newly formed cementum and alveolar bone, reconstructing periodontal support tissue [1]. However, the periodontium consists of many different tissues with various healing abilities. Integral regeneration of the periodontium using different periodontal treatment has been difficult to achieve.

The present strategy of periodontal tissue regeneration includes eliminating the inflammatory process, arresting disease progression, and ultimately regenerating the lost periodontal structures. Recent studies have indicated that periodontal ligament stem cells (PDLSCs) are the most promising endogenous seed cells for periodontal tissue regeneration [2–5]. PDLSCs have a favourable effect on newly formed bone and newly formed periodontal ligament compared to other stem cell sources [6]. However, PDLSCs function is impaired in patients with periodontitis due to the long-term inflammation. Compared with healthy PDLSCs, proliferation and migration of PDLSCs isolated from the inflamed periodontium could be maintained, whereas the multipotency of the inflamed cells in vitro was obviously damaged along with the involvement of these cells for tissue regeneration in vivo [7]. Both canonical Wnt/β-catenin signalling pathway and the noncanonical Wnt/Ca^2+^ signalling pathways play a role in the impaired osteogenic differentiation of PDLSCs, particularly under chronic inflammation [8]. Further, immunomodulatory properties of inflamed PDLSCs were markedly dysfunctional [9]. Therefore, it is extremely important to repair the self-renewal and differentiation abilities of PDLSCs.

Dental follicle stem cells (DFSCs) are mesenchymal stem cell (MSC)-like progenitor cells present in the dental follicle and thus are closely related to PDLSCs developmentally and functionally [10]. In the inflamed periodontal microenvironment, DFSCs were able to enhance the proliferation as well as the osteogenic and adipogenic differentiation of both PDLSCs and inflamed PDLSCs to different degrees. Moreover, when co-cultured with DFSCs, cell layers and extracellular matrix of PDLSCs/inflamed PDLSCs cell sheets increased in vitro and periodontal regeneration improved in vivo [11]. In our previous study, periodontal tissue-like structures were successfully observed in the periodontal bone defect zone in rats after transplantation of DFSCs sheets alone or in combination with scaffold [12]. These findings demonstrated that DFSCs had positive effects on periodontal tissue regeneration. Although the multipotent ability of MSCs has already been proven, researchers have realized that only a small portion of the cells could be engrafted into local organs or tissues compared to the large numbers of transplanted cells. Hence, the mechanisms underlying the function of DFSCs in periodontal tissue regeneration remain to be elucidated. Additionally, questions on whether the differentiation or the paracrine effect of MSCs is the leading mechanism of tissue regeneration have been raised.

The paracrine pathway is a dominant mechanism through which MSCs promote tissue repair and regeneration [13]. A variety of soluble proteins such as cytokines and growth factors can be secreted by MSCs, which thereafter modulate immune response; suppress fibrosis, oxidative stress, and apoptosis; and enhance self-renewal and differentiation. Moreover, a category of specific extracellular vesicles sized less than 200 nm that serve as an efficient mechanism in cell-to-cell communication can be also secreted by the MSCs and are known as the small extracellular vesicles (sEVs) [14]. sEVs play a crucially effective role by carrying and transferring important bioactive molecules such as proteins, lipids, and nucleic acids among cells [15]. Nowadays, with the increasing attention paid to cell-free therapy in tissue regeneration, it is apparent that the transplantation of sEVs harbours the potential to avoid a series of immune rejection caused by the cells obtained from different donors.

Increasing evidence has proved that sEVs derived from MSCs could promote the regeneration of skin [16, 17], bone [18, 19], and cartilage [20, 21]. In parallel, the potential clinical application of sEVs derived from dental stem cell in dental tissue regeneration has been shared [22]. DFSCs conditioned medium was able to provide a favourable microenvironment for self-renewal and osteogenesis of PDLSCs [11]. Nevertheless, as an important intermediate carrier in cell-to-cell communication, it is unclear whether sEVs are the central factor contributing to promote the proliferation and differentiation ability of PDLSCs and its potential application value as a novel cell-free strategy for periodontal regeneration.

In this study, we explored the effects of sEVs derived from DFSCs (DFSCs-sEVs) on the proliferation, migration, and osteogenic differentiation of PDLSCs in vitro. Furthermore, we studied the effects of DFSCs-sEVs on periodontal tissue repair in a rat model in vivo*.* Evidence regarding the validity and feasibility of DFSCs-sEVs-based treatment for future clinical periodontal tissue regeneration has been presented.

## Methods

### Isolation and characterization of DFSCs and PDLSCs

This study was approved by the Research Ethics Committee of Hospital of Stomatology, Kunming Medical University (Project No. KYKQ2020ME C020). All patients provided written informed consent. Dental follicles were obtained from immature third molars extracted for impaction of young patients (aged 17–22 years-old). Periodontal ligaments were collected from healthy premolars extracted for orthodontic treatment. The harvested tissues were separated and rinsed with PBS (Beyotime, Shanghai, China) three times. Dental follicles were cut into pieces, while periodontal ligament tissues were scraped from the middle 1/3 of the root with a scalpel. Next, the tissues were digested using collagenase type I (Sigma-Aldrich, St. Louis, MO, USA) for 20 min and incubated in DMEM/F12 (Biological Industries, Israel) medium containing 20% foetal bovine serum (FBS, Gibco, Invitrogen, Carlsbad, Calif, USA) at 37 °C under 5% CO_2_. The media was changed every 3 days, and the cells of passage 3–5 were used for the experiment.

After three passages, the cells were harvested for identification. For osteogenic and adipogenic differentiation, the cells were cultured in 6‐well plates (1 × 10^5^ cells/well). After reaching 80% confluency, the cells were orientiatedly induced using osteogenic differentiation medium (Cyagen, Suzhou, China) and adipogenic differentiation medium (Cyagen, Suzhou, China) for 3 weeks, respectively. After differentiation, cells were stained with Alizarin red or Oil Red O and observed under an inverted microscope (OLYMPUS CKX53, Tokyo, Japan). Immunofluorescence staining was used to detect cell surface markers. Briefly, cells were inoculated at 1 × 10^5^ cells/well on a six-well. At 80% confluence, cells were fixed with 4% paraformaldehyde, washed with PBS, and stained with the following antibodies: anti-Vimentin (1:100, Zen-Bio, Chengdu, China), anti-Nestin (1:25, Zen-Bio, Chengdu, China), anti-CD146 (1:100, Abcam, Wales, UK) according to the manufactures' protocols, then visualised with Fluorescein-Conjugated Goat anti-Rabbit IgG (1:250, ZSGB-Bio, Beijing, China). 6-diamidino-2-phenylindole (DAPI, Beyotime, Shanghai, China) solution was used for the staining of nuclei. Images were obtained using a laser scanning confocal microscope (Nikon, Tokyo, Japan). For flow cytometric analysis, the cells were resuspended in cold PBS containing 2% FBS at a concentration of 1 × 10^6^ cells/mL prior to adding the following monoclonal antibodies: CD29-PE, CD44-PE, CD90-PE, CD105-PE, CD34-FITC, and CD45-FITC (BD Biosciences, USA). The unmarked cells were used as a negative control. Finally, the stained cells were analysed using BD Accuri® C6 (Beckman-coulter, Bria, California, USA) and FloMax® software. Cell clone formation ability was tested using plate clone formation assay.

### Isolation of DFSCs-sEVs

When cell reached 80% confluency, DFSCs were cultured using the Exo-Clear™ Complete Cell Growth Medium (System Biosciences, Calif, USA) for 48 h, and the supernatant was collected and concentrated with a 3 kDa ultrafiltration tube (Millipore, USA) at 5,000 × *g* for 30 min at 4 °C. sEVs were extracted from the concentrated supernatant using the exoEasy Maxi Kit (QIANGEN, Germany) according to the manufacturer’s protocol. The protein concentration of DFSCs-sEVs was determined using the BCA Protein Assay Kit (Beyotime).

### Scanning electron microscope (SEM)

PP3010 SEM Cryo System (Quorum Technologies, England) was used to cool the sample table to below − 190 °C. Next, 10 μg DFSCs-sEVs, collagen sponges, and collagen sponges loaded with DFSCs-sEVs were placed on the sample table. Images of the particles were captured using a scanning electron microscope (Hitachi High-Tech, Suzhou, China).

### Nanoparticle tracking analysis (NTA)

Extracted DFSCs-sEVs samples were diluted in ddH_2_O to a concentration of 10^7^ to 10^9^ particles/mL. The number and size of particles in the samples were measured using Zetaview PMX110 (Particle Metrix, Germany) with a 405 nm laser. Photographs were taken at 30 pictures per s, lasting for 1 min. The movement of particles was analysed using the NTA software (ZetaView8.02.28).

### PDLSCs treated with DFSCs-sEVs

In the proliferation and migration assays, when PDLSCs successfully adhered, the medium was changed to a serum-free medium overnight. Subsequently, PDLSCs were grouped based on the following treatments: (1) sEVs-Clear medium (Control); (2) sEVs-Clear medium with 10 µg/mL DFSCs-sEVs; (3) sEVs-Clear medium with 50 µg/mL DFSCs-sEVs; (4) sEVs-Clear medium with equal volume PBS.

During 14 days of osteoinduction, PDLSCs were treated with DFSCs-sEVs.

### DFSCs-sEVs uptake assay

DFSCs-sEVs were labelled with PKH26 (Sigma-Aldrich) according to the manufacturer's instruction. Briefly, 10 μg DFSCs-sEVs was diluted in 1 mL Diluent C containing 6 μL PKH26 dye for 5 min. Ten millilitres of 5% bovine serum albumin (BSA, Beyotime) was added to bind excess dye. The labelled DFSCs-sEVs were concentrated with ultrafiltration tube at 4 °C, 5,000 × *g* for 30 min. After incubation with labelled DFSCs-sEVs for 4 h or 24 h, PDLSCs were washed with PBS, fixed with 4% paraformaldehyde, stained with DAPI, and then observed under a laser scanning confocal microscope (Nikon).

### Proliferation assay

PDLSCs were seeded in a six-well plate (1 × 10^5^ cells/well) and in a 96-well plate (5 × 10^3^ cells/well). After starvation overnight, the cells were treated with 10 and 50 µg/mL of DFSCs-sEVs, respectively. At 24 h after DFSCs-sEVs stimulation, EdU staining (Ribo-Bio, Guangzhou, China) was performed, and cells were imaged using a laser scanning confocal microscope; the result was analysed manually using the ImageJ software (NIH, Bethesda, MD). Cell cycle analysis (MeilunBio, Dalian, China) was performed according to the manufacturer's instruction. The stained cells were analysed on an Agilent NovoCyte Fluidics Station (Agilent Technologies, Santa Clara, Calif, USA) at 24 and 48 h after DFSCs-sEVs treatment. The data were analysed using GraphPad Prism 8 (GraphPad Software, USA). At 24, 48, and 72 h after DFSCs-sEVs treatment, cell counting kit-8 (CCK-8, MeilunBio) was used to measure the absorbance at 450 nm with a microplate reader (Thermo, USA).

### Migration assay

PDLSCs were seeded into a 24-well plate at a density of 1 × 10^5^ cells/well with a Culture-Insert (Ibidi, Martin Redd, Germany) placed at the centre of each well. Scratches having a width of 500 μm were formed upon removing the insert the next day. After washing thrice with PBS, cells were cultured in a serum-free medium. At 0, 12, and 24 h after DFSCs-sEVs treatment, the same position was photographed using an inverted microscope (OLYMPUS CKX53). The width of the scratches was measured manually using the ImageJ software, and the ratio of migration was calculated.

### Osteogenic differentiation assay

PDLSCs were seeded in six-well plates (1 × 10^5^ cells/well). When the cells reached 80% confluence, the culture medium was replaced with different osteogenic medium (Supplemented with 10 μg/mL DFSCs-sEVs or PBS). The solution was changed every 3 days. After 14 days of induction, alizarin red staining was performed to observe the formation of mineralised nodules. The expression of osteogenic mRNA and proteins (COL1, ALP, RUNX2, BSP) was detected at gene level (qRT-PCR) and protein level (Western blot) of the samples at day 7 and 14 of intervention culture.

### Quantitative reverse transcription polymerase chain reaction (qRT-PCR)

According to the manufacturer's protocol, total RNA was isolated using the TaKaRa MiniBEST Universal RNA Extraction Kit (Takara, Osaka, Japan). Total RNA was reverse transcribed into cDNA using the PrimeScript™ RT Master Mix (Perfect Real Time) (Takara), and qRT-PCR was conducted using a QuantStudio™ Real-Time PCR System (Thermo Fisher Scientific, USA) with the TB Green® Premix Ex Taq™ II (Tli RNaseH Plus) (Takara). Relative expression levels were calculated using the 2^−ΔΔCt^ method and determined by normalizing to the values of glyceraldehyde 3-phosphate dehydrogenase (*GAPDH*). The primers were synthesized by Sangon Biotech (Table 1, China).

**Table 1:** List of primer sequences

Gene	Forward primer (5′–3′)	Reverse primer (5′–3′)
*COL1*	AACATGGAGACTGGTGAGACCT	CGCCATACTCGAACTGGAATC
*ALP*	TAAGGACATCGCCTACCAGCTC	TCTTCCAGGTGTCAACGAGGT
*RUNX2*	CTTTACTTACACCCCGCCAGTC	AGAGATATGGAGTGCTGGTC
*BSP*	CGAACAAGGCATAAACGGCACCAG	TTCTCCATTGTCTCCTCCGCTGCT
*CDKN1C*	CAGAACCGCTGGGATTACGACTTC	TCGCTGTCCACTTCGGTCCAC
*FGF7*	TGACATGGATCCTGCCAACTTTGC	GCTCAGGGCTGGAACAGTTCAC
*SOD2*	CCCGACCTGCCCTACGACTAC	AACGCCTCCTGGTACTTCTCCTC
*CRISPLD2*	GACTGCTACACGACCGTTGCTC	GCCCAGTAGGAAGGTTCGTCTTTG
*TRIB3*	CCACCGTATCCCTGAGCCTGAG	AGCGAAGACAAAGCGACACAGC
*SOCS1*	CCAGGTGGCAGCCGACAATG	CGAGGAGGAGGAAGAGGAGGAAG
*SLC20A1*	AGCGTGGACTTGAAAGAGGAAACC	TTGCTGACGGCTTGACTGAACTG
*EGR3*	CACCACTCACATCCGCACTCATAC	TTCTCCGCCTTCTTCTCCTTTTGC
*GAPDH*	CTTTGGTATCGTGGAAGGACTC	GTAGAGGCAGGGATGATGTTCT

ALP, alkaline phosphatase; BSP, bone sialoprotein; CDKN1C, cyclin-dependent kinase inhibitor 1C; COL1, collagen1; CRISPLD2, cysteine-rich secretory protein LCCL domain containing 2; EGR-3, early growth response-3; FGF7, fibroblast growth factor 7; GAPDH, glyceraldehyde 3-phosphate dehydrogenase; RUNX2, runt-related transcription factor 2; SOCS1, suppressor of cytokine signalling 1; SLC20A1, solute carrier family 20 member 1; SOD2, superoxide dismutase 2; TRIB3, tribbles pseudokinase 3

### Western blotting

Western blotting was performed as previously described [10]. Briefly, PDLSCs and DFSCs-sEVs were lysed with radioimmuno-precipitation assay lysis buffer (Solarbio, Beijing, China). The protein levels were quantified using a Bradford assay kit (Beyotime). Antibodies against CD81 (#ab79559), TSG101 (#ab125011), BSP (#ab125227), RUNX2 (#ab92336), ALP (#ab95462), and COL1 (#ab90395) were purchased from Abcam (Wales, UK). Antibodies against HSP90 (#TA500494) and β-actin (#TA811000) were purchased from OriGene Technologies (Rockville, USA). Antibodies against p38 MAPK (#8690T), ERK1/2 (#4695T), Phospho-p38 MAPK (#4511T), and Phospho-ERK1/2 (#4370T) were purchased from Cell Signaling Technology.

### RNA sequencing and functional enrichment analysis

PDLSCs from 3 different donors were cultured in serum-free medium supplemented with 10 μg/mL DFSCs-sEVs. After 3 days, rRNAs were removed from total RNAs using the Epicentre Ribo-Zero rRNA removal kit (illumine, San Diego, California, USA), and RNA-seq libraries were generated and indexed using the NEBNext Ultra RNA Library Prep Kit (New England Biolabs, Ipswich, MA, USA). A 150-bp-paired-end sequencing was performed using the Annoroad Genomics and Illumina HiSeq 3000 system by RiboBio (Guangzhou RiboBio Co., Ltd.). The sequencing reads were filtered and qualified using the Trimmomatic tools (v 0.36), and pseudo-aligned to the reference genome using HISAT2. The differential gene expression analysis was performed using DEGseq under the condition of P-value threshold of < 0.05 and |log2(fold change)|> 1. Further analysis using the Gene Ontology (GO) database and the Kyoto Encyclopedia of Genes and Genomes (KEGG) pathway database were conducted using gplots package in R software.

### Inhibition of MAPK signalling pathway

The suitable concentration of inhibitor U0126 (ERK1/2 signalling pathway inhibitor, Cell Signaling Technology, Boston, USA) and SB202190 (p38 MAPK signalling pathway inhibitor, Absin, Shanghai, China) were selected based on the previous literature [23]. After stimulation with U0126 or SB202190 for 2 h, PDLSCs were treated with 10 μg/mL DFSCs-sEVs for 15 and 60 min and 24, 48, and 72 h.

At 24 h after DFSCs-sEVs stimulation, EdU staining was performed, and the cells were photographed under a laser scanning confocal microscope. The result was analysed manually using the ImageJ software. At 24, 48, and 72 h after DFSCs-sEVs stimulation, CCK-8 assay was implemented to detect the proliferation of PDLSCs.

### Rat periodontal defect model

All animal experiments in this study were approved and conducted in accordance with the guidelines of the Animal Experiment Ethics Review Committee of Kunming Medical University (Approval No. KMMU 2021011). A total of 72 12-week-old male Sprague–Dawley (SD) rats were utilized in this study. Animals were allowed free access to water and food and were kept in a controlled environment (50% humidity, 25 °C, and 12-h light–dark cycle).

All animals were randomly divided into 3 groups: blank group (Untreated, *n* = 24); collagen sponges (ZH-Bio, Chengdu, China) group (CS, *n* = 24); collagen sponges group loaded with DFSCs-sEVs (CS-sEVs, *n* = 24). Periodontal defect (3 × 2 × 1 mm) was inflicted on the periodontal tissue of the first molar in rats as previously reported [24]. The rats were treated according to the groups, and mandibular samples were collected at 2, 4, and 8 weeks after surgery.

### Localization of DFSCs-sEVs in the periodontal defect area

DFSCs-sEVs labelled with PKH26 were loaded on collagen sponges and transplanted into the periodontal defect area of SD rats. Samples were collected 2 and 4 weeks after surgery. The samples were fixed using 4% paraformaldehyde for 24 h, decalcified (Maxim-Bio, Fuzhou, China) for 1 week and dehydrated using a graded series of sucrose. Subsequently, the samples were embedded in optimal cutting temperature compound (O.C.T Compound), and frozen sections were prepared. Anti-fluorescence quenching sealing solution was used to seal the sections. The images were obtained using a laser scanning confocal microscope, and the result was calculated manually using the ImageJ software.

### Micro-CT analysis

The collected samples were fixed using 4% paraformaldehyde for 24 h. Next, a NEMO® NMC-100 micro-CT system (PINGSENG Healthcare Inc., Kunshan, China) was used to scan the submaxillary molar regions with the following parameters: 90 kV source voltage and 60 μA source current. The data were reconstructed, and the bone volume/tissue volume (BV/TV) ratio and the trabecular thickness (Tb. Th) of the defect area were analysed using the Avatar 1.5.0 software (PINGSENG Healthcare Inc.).

### Histological analysis

The samples were fixed using 4% paraformaldehyde for 24 h, decalcified for 1 week, dehydrated using a graded series of ethanol, and embedded in paraffin wax. Embedded sections were prepared for H&E staining.

### Statistical analysis

All experimental data were statistically analysed using SPSS 19.0. Independent sample t-test was used for comparison between two groups, and one-way ANOVA was used for comparing 3 or more groups. Statistical significance was set at *p* < 0.05.

## Results

### Characteristics of cells and DFSCs-sEVs

**Fig. 1:**
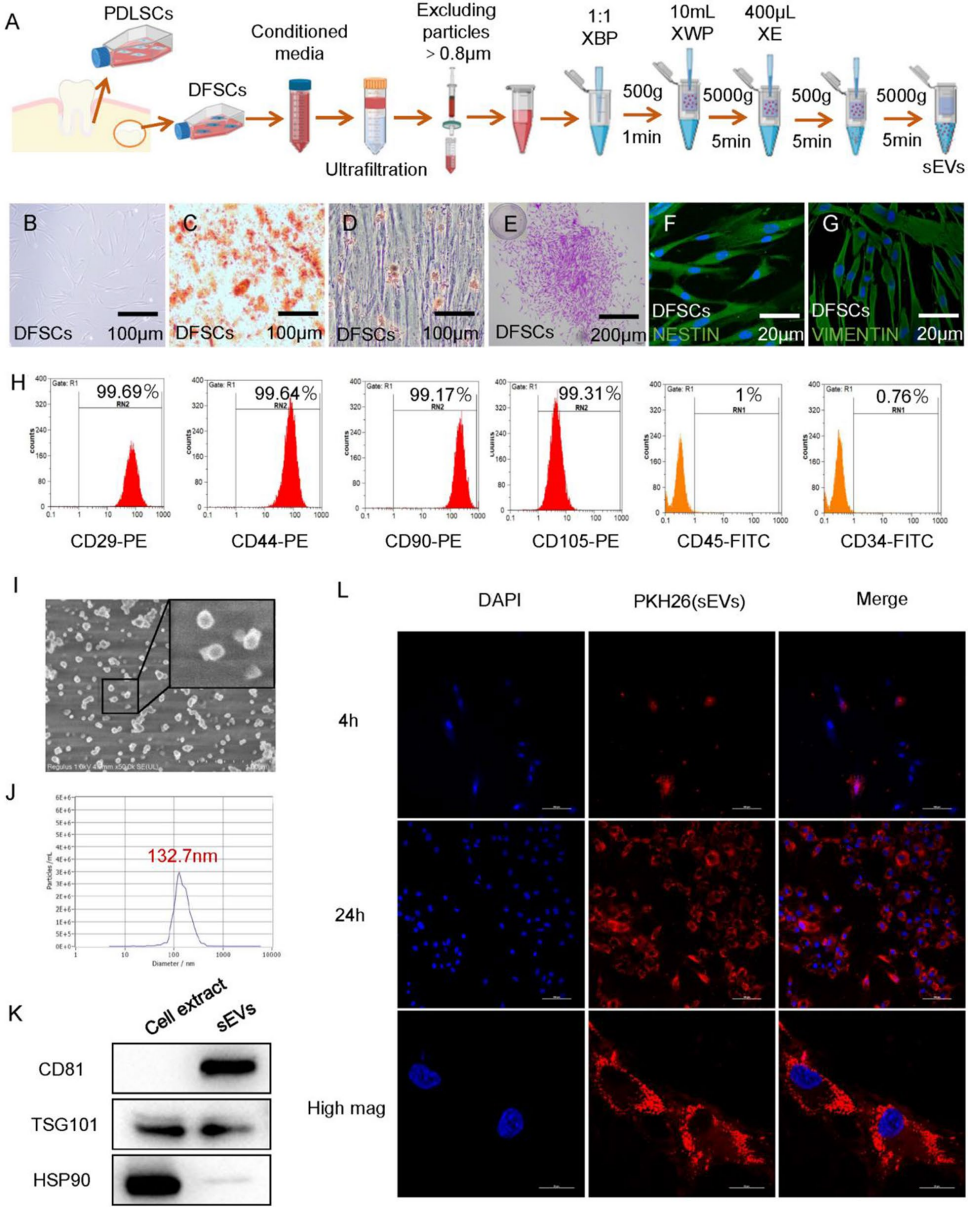
Characteristics of DFSCs-sEVs. **A** Flowchart of cells and DFSCs-sEVs. **B** DFSCs at passage 3 exhibited the typical long spindle shape. **C** Osteogenesis ability of DFSCs was detected by Alizarin red staining. **D** Adipogenesis ability of DFSCs was detected by Oil red O staining. **E** Colony formation of DFSCs. **F** Nestin expression in passage 3 DFSCs. **G** Vimentin expression in passage 3 DFSCs. **H** Flow cytometric analysis of surface markers in DFSCs, CD29-PE (99.69%), CD44-PE (99.64%), CD90-PE (99.17%), CD105-PE (99.31%), CD34-FITC (0.76%) and CD45-FITC (1%). **I** Morphology of sEVs observed using scanning electron microscopy. **J** Particle size distribution of sEVs measured using ZetaView analysis. **K** sEVs surface markers CD81, TSG101 and HSP90 detected by western blotting. The samples derive from the same experiment and that blots were processed in parallel. Full-length gel images are provided in the supplementary file. **L** Cellular internalization of sEVs by PDLSCs. The nucleus of PDLSCs was stained with DAPI (blue), and sEVs were labelled with PKH26 (red). Scale bar = 100 µm. Scale bar of high mag = 20 μm

DFSCs and PDLSCs exhibited the typical long spindle shape or fusiform (Fig. 1B and Additional file 1: S1A). Cells were differentiated into osteogenic (Fig. 1C and Additional file 1: S1B) and adipogenic (Fig. 1D and Additional file 1: S1C) cells and formed colonies (Fig. 1E and Additional file 1: S1D). In addition, cells were identified by the surface markers nestin ( +) (Fig. 1F), vimentin (+) (Fig. 1G and Additional file 1: S1E), and CD146(+) (Additional file 1: Figure S1F) using immunofluorescence staining and CD29-PE (99.69%/99.85%), CD44-PE (99.64%/99.65%), CD90-PE (99.17%/ 99.33%), CD105-PE (99.31%/ 98.53%), CD45-FITC (1%/0.98%) and CD34-FITC (0.76%/1%) using flow cytometry (Fig. 1H and Additional file 1: S1G).

SEM images revealed that DFSCs-sEVs showed a typical saucer-like shape (Fig. 1I). NTA analysis indicated that the average diameter of most DFSCs-sEVs was 132.7 nm (Fig. 1J). Western blotting confirmed that DFSCs-sEVs expressed CD81, TSG101, and HSP90 (Fig. 1K). This finding was consistence with a previous report [25]. To determine the uptake of DFSCs-sEVs by PDLSCs, we incubated the PKH26-labelled DFSCs-sEVs with PDLSCs. After PDLSCs were treated with DFSCs-sEVs for 4 and 24 h, PKH26-labelled DFSCs-sEVs (red dots) were gradually internalised by PDLSCs (Fig. 1L).

### DFSCs-sEVs promoted the proliferation and migration of PDLSCs

**Fig. 2:**
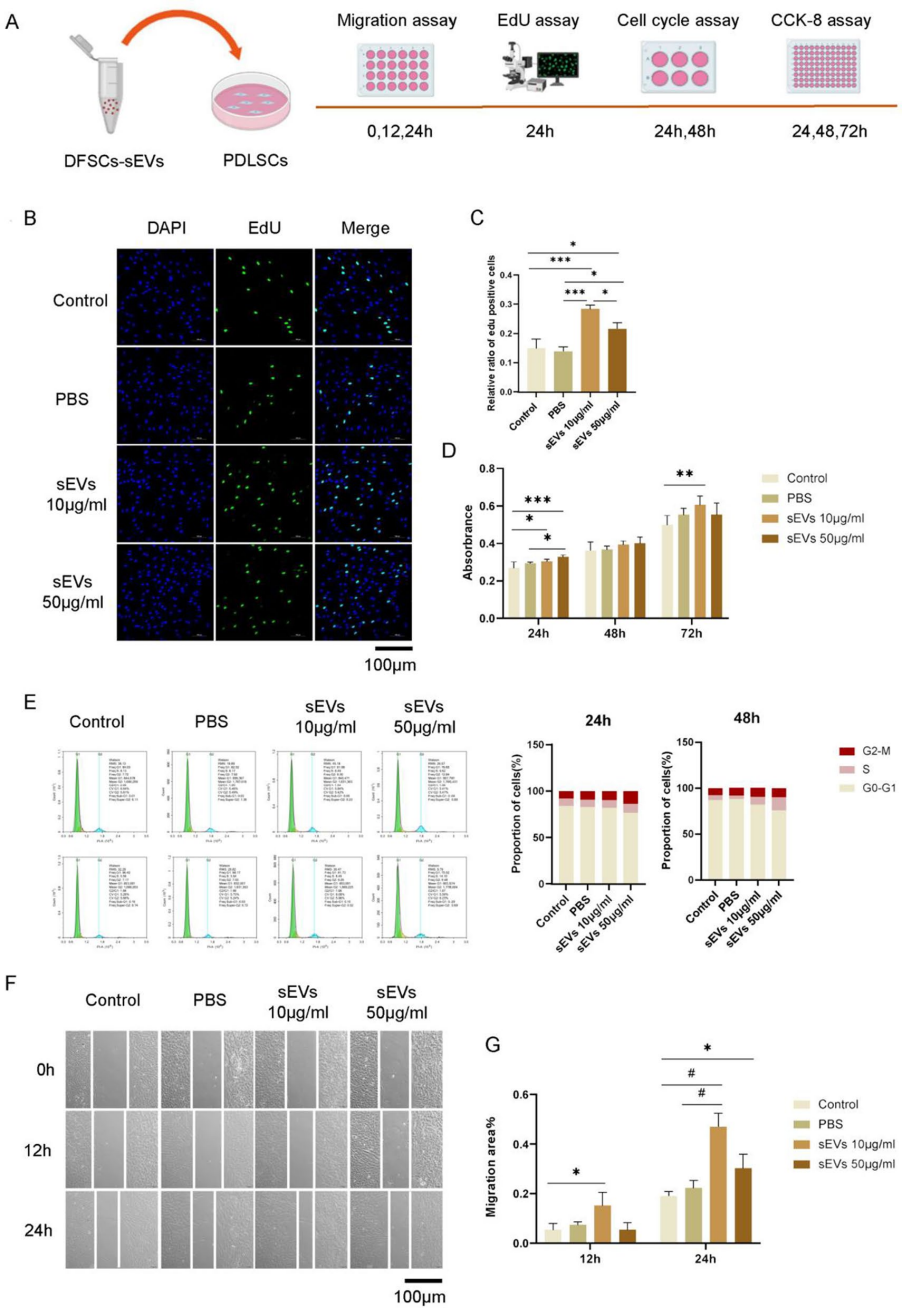
DFSCs-sEVs promoted proliferation and migration of PDLSCs. (A) Experiment design for proliferation and migration assays. (B, C) The effect of DFSCs-sEVs on PDLSCs proliferation detected using EdU assay, and quantification of EdU positive cells. Scale bar = 100 μm. (D) CCK-8 assay detected the effect of DFSCs-sEVs on PDLSCs proliferation. (E) Cell cycle assay showed DFSCs-sEVs enhanced the proportion of S phase and G2 phase of PDLSCs. (F) Wound healing assay detected the effect of DFSCs-sEVs on PDLSCs migration. Scale bar = 100 μm. (G) Quantitative analysis of migration rates. *n* = 3, *p < 0.05; **p < 0.01; ***p < 0.001; #*p* < 0.0001

EdU staining and CCK-8 showed that DFSCs-sEVs enhanced proliferation of PDLSCs (Fig. 2B–D). Consistently, cell cycle analysis also revealed that both the 10 μg/mL DFSCs-sEVs group and the 50 μg/mL DFSCs-sEVs group displayed a significantly higher percentage of PDLSCs in the S and G2 phases at 24 and 48 h (Fig. 2E). Moreover, PDLSCs treated with 10 μg/mL DFSCs-sEVs had a stronger proliferative ability at 72 h (Fig. 2D).

Wounding healing assay showed that 10 μg/mL DFSCs-sEVs significantly enhanced the migration of PDLSCs at 12 and 24 h (Fig. 2F–G). Therefore, 10 μg/mL DFSCs-sEVs was chosen for follow-up experiments.

### DFSCs-sEVs facilitated osteogenic differentiation of PDLSCs

**Fig. 3:**
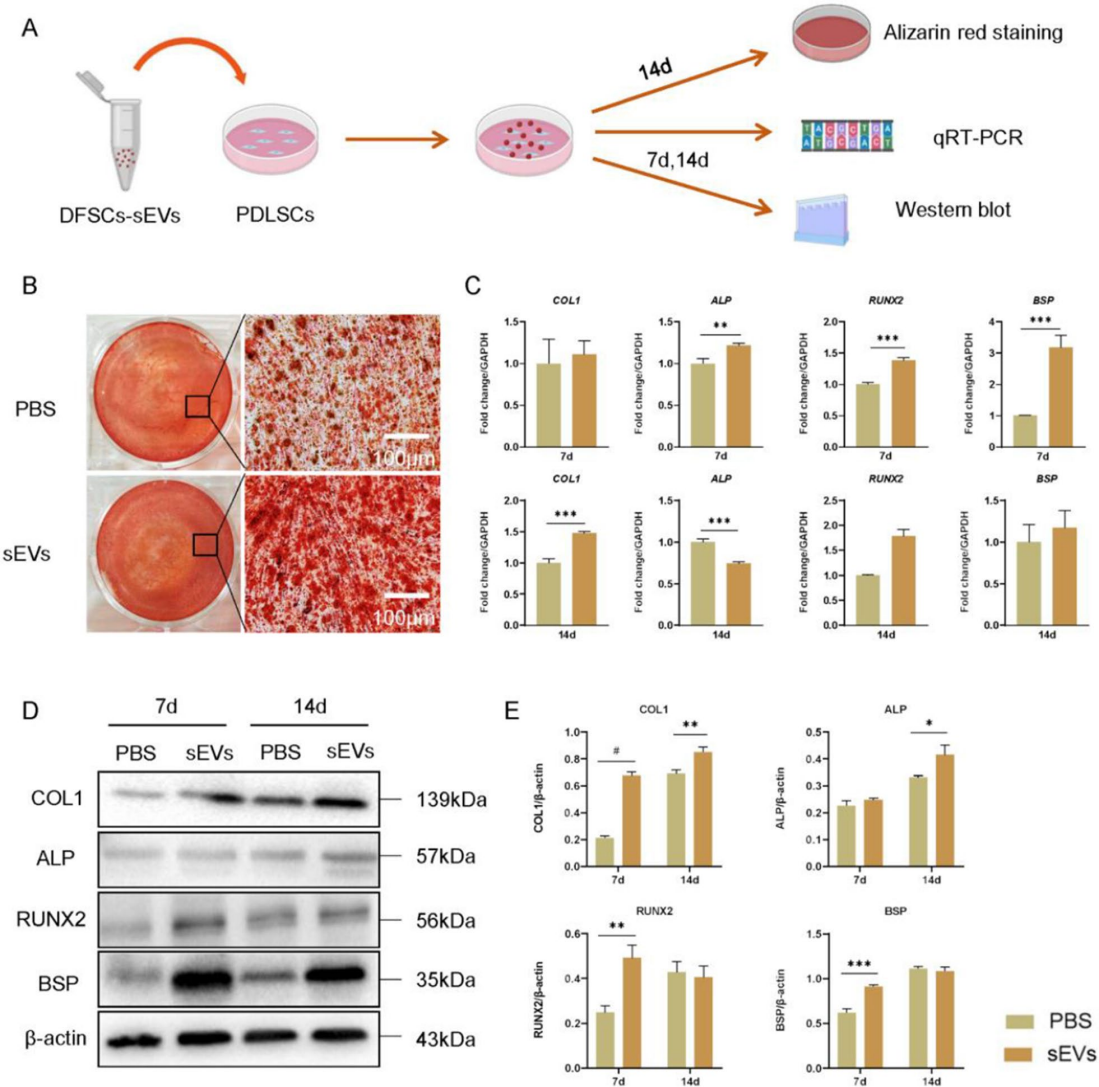
DFSCs-sEVs facilitated osteogenic differentiation of PDLSCs. **A** Experiment design for osteoinduction. **B** Alizarin red staining of PDLSCs on day 14 after osteoinduction showed that more mineralized nodules formed in the PDLSCs treated with DFSCs-sEVs. Scale bar = 100 μm. **C** Expression of osteogenic genes *COL1*, *ALP*, *RUNX2*, and *BSP* in PDLSCs for 7 and 14 days after osteoinduction. **D** Expression of osteogenic proteins COL1, ALP, RUNX2, and BSP in PDLSCs for 7 and 14 days after osteoinduction. **E** Quantitative analysis of protein expression levels. The samples derive from the same experiment and that blots were processed in parallel. Full-length gel images are provided in the supplementary file.*n* = 3, *p < 0.05; **p < 0.01; ***p < 0.001; #*p* < 0.0001

Alizarin red staining showed that more mineralized nodules formed in the DFSCs-sEVs group than in the PBS group at 14 days after induction (Fig. 3B). qRT-PCR also revealed that the expression of *ALP*, *RUNX2* and *BSP* increased in the DFSCs-sEVs group at 7 days after induction, while *COL1* was upregulated in the DFSCs-sEVs group at 14 after induction (Fig. 3C). Western blot showed that novel markers of osteogenic differentiation, including COL1, RUNX2, and BSP were higher in the DFSCs-sEVs group at 7 days after induction, while COL1 and ALP were upregulated in the DFSCs-sEVs group at day 14 after induction (Fig. 3D–E).

### DFSCs-sEVs promoted the proliferation of PDLSCs through the p38 MAPK signalling pathway

**Fig. 4:**
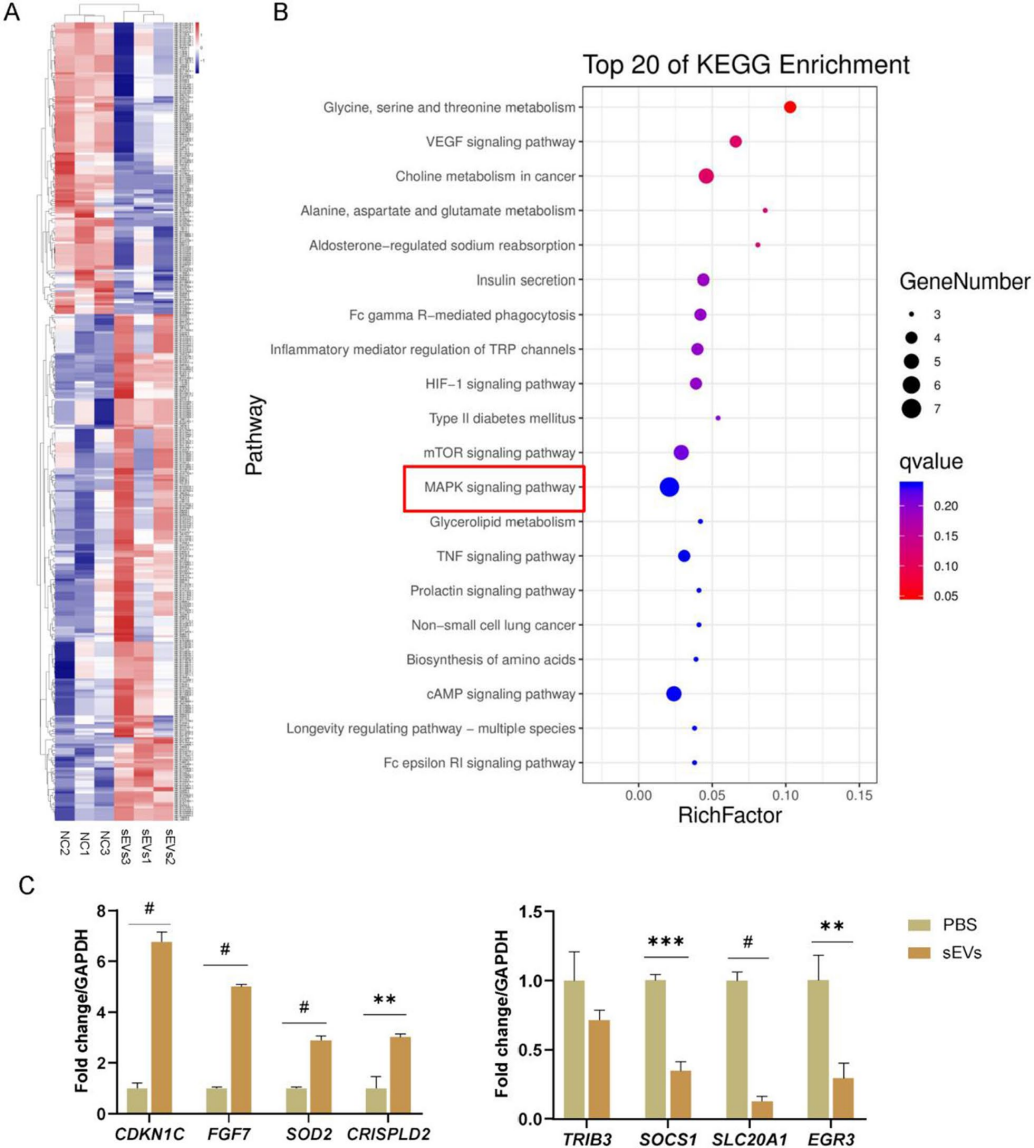
Transcriptome sequencing data. **A** Heat map depicting gene expression changes in DFSCs-sEVs-treated and -untreated PDLSCs. **B** Kyoto Encyclopedia of Genes and Genomes (KEGG) classification for differently expressed genes in DFSCs-sEVs-treated and -untreated PDLSCs. **C** The results of transcriptome sequencing were verified using qRT-PCR. *n* = 3, *p < 0.05; **p < 0.01; ***p < 0.001; #*p* < 0.0001

Differentially expressed genes between the DFSCs-sEVs treated group and the control group were depicted using a heat map (Fig. 4A). GO analysis of these genes suggested that DFSCs-sEVs might be involved in regulating the proliferation of PDLSCs (Additional file 1: Figure S2). Next, the KEGG pathways enrichment analysis was performed to elucidate the significant enrichment pathways with top 20 enrichment score values in the sEVs-treated PDLSCs. The results showed that the differentially expressed genes were significantly enriched in the MAPK signalling pathway (Fig. 4B). Therefore, four upregulated and downregulated genes were selected separately for qRT-PCR verification. qRT-PCR showed that expression of these RNA-seq was consistent with the sequencing results (Fig. 4C).

**Fig. 5:**
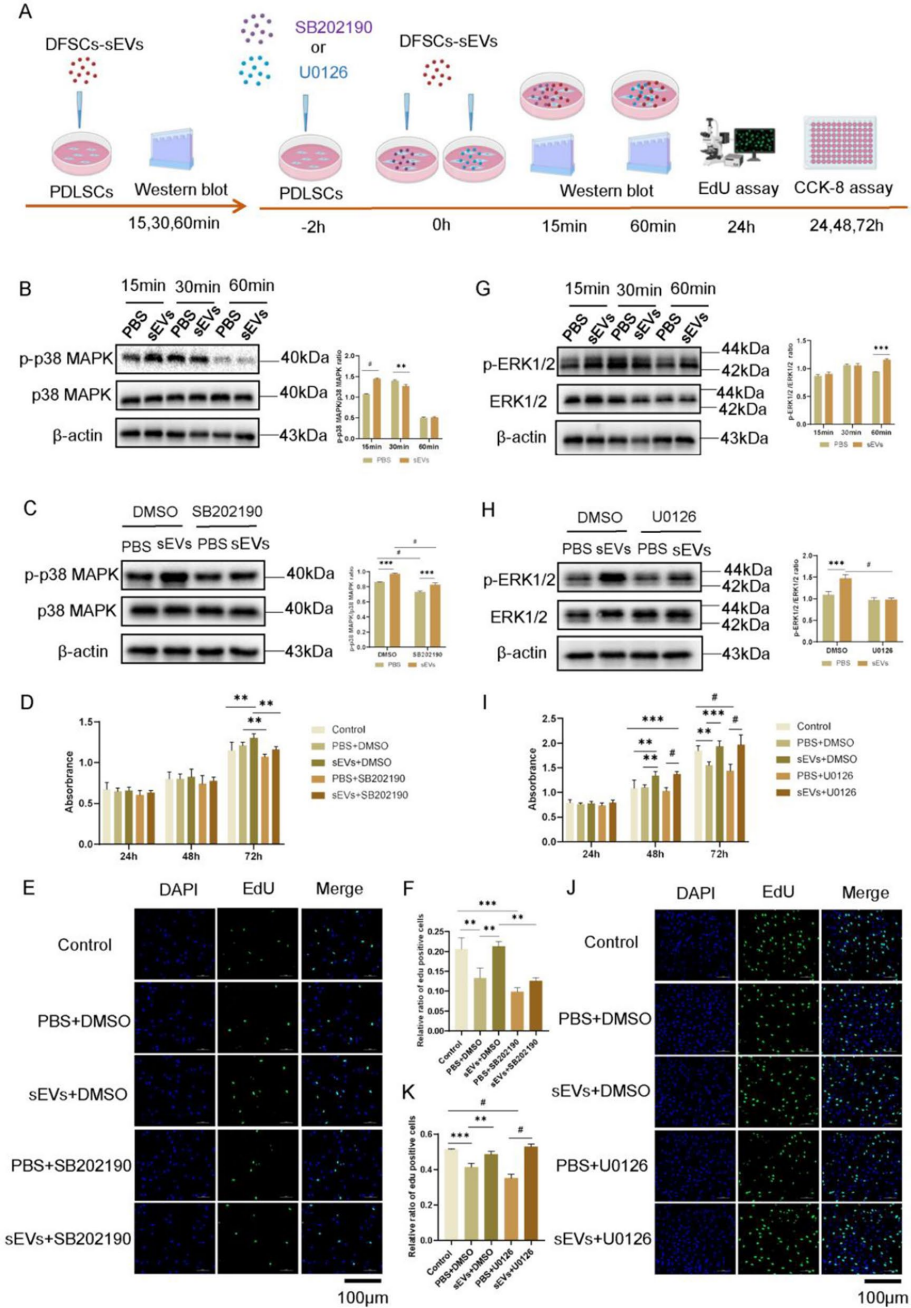
DFSCs-sEVs promoted proliferation of PDLSCs through the p38 MAPK signalling pathway. **A** Time schedule for treatment of PDLSCs in vitro. **B**, **G** Western blot analysis detected the phosphorylation of p38 MAPK and ERK1/2 signalling pathways in PDLSCs following treatment with DFSCs-sEVs, and quantitative analysis of protein expression levels. **C**, **H** Western blot analysis detected the phosphorylation of p38 MAPK and ERK1/2 signalling pathways in PDLSCs following treatment with the p38 MAPK inhibitor SB202190 or the ERK1/2 inhibitor U0126. The samples derive from the same experiment and that blots were processed in parallel. Full-length gel images are provided in the supplementary file. **D**, **I** CCK-8 assay detected the effect of DFSCs-sEVs on PDLSCs proliferation following treatment with the MAPK inhibitor SB202190 or the ERK1/2 inhibitor U0126. (E, F) EdU assay detected the effect of DFSCs-sEVs on PDLSCs proliferation following treatment with the p38 MAPK inhibitor SB202190, and quantification of EdU positive cells. Scale bar = 100 μm. **J**, **K** EdU assay detected the effect of DFSCs-sEVs on PDLSCs proliferation following treatment with the ERK1/2 inhibitor U0126, and quantification of EdU positive cells. Scale bar = 100 μm. *n* = 3, *p < 0.05; **p < 0.01; ***p < 0.001; #*p* < 0.0001

As shown via western blotting, p38 MAPK was activated at 15 min post DFSCs-sEVs treatment, while ERK1/2 was activated at 60 min post treatment (Fig. 5B, G). We observed that when p38 MAPK signalling was inhibited by SB202190, the DFSCs-sEV-mediated activation of p38 MAPK was impaired, while there was no obvious change when the ERK1/2 pathway was inhibited using U0126. This indicated that when p38 MAPK/ERK1/2 signalling pathway was inhibited, DFSCs-sEVs could partly rescue the activation of p38 MAPK signalling pathway (Fig. 5C); however, there were no obvious effects of DFSCs-sEVs on the activation of the ERK1/2 signalling pathway (Fig. 5H). We also observed that SB202190 significantly reduced the proliferation of PDLSCs promoted by DFSCs-sEVs (Fig. 5D–F), while there were no significant effects of U0126 on the proliferation of PDLSCs treated with DFSCs-sEVs (Fig. 5I–K). These results demonstrated that DFSCs-sEVs might promote the proliferation of PDLSCs partly by activating the p38 MAPK signalling pathway.

### DFSCs-sEVs promoted periodontal tissue regeneration in rats

**Fig. 6:**
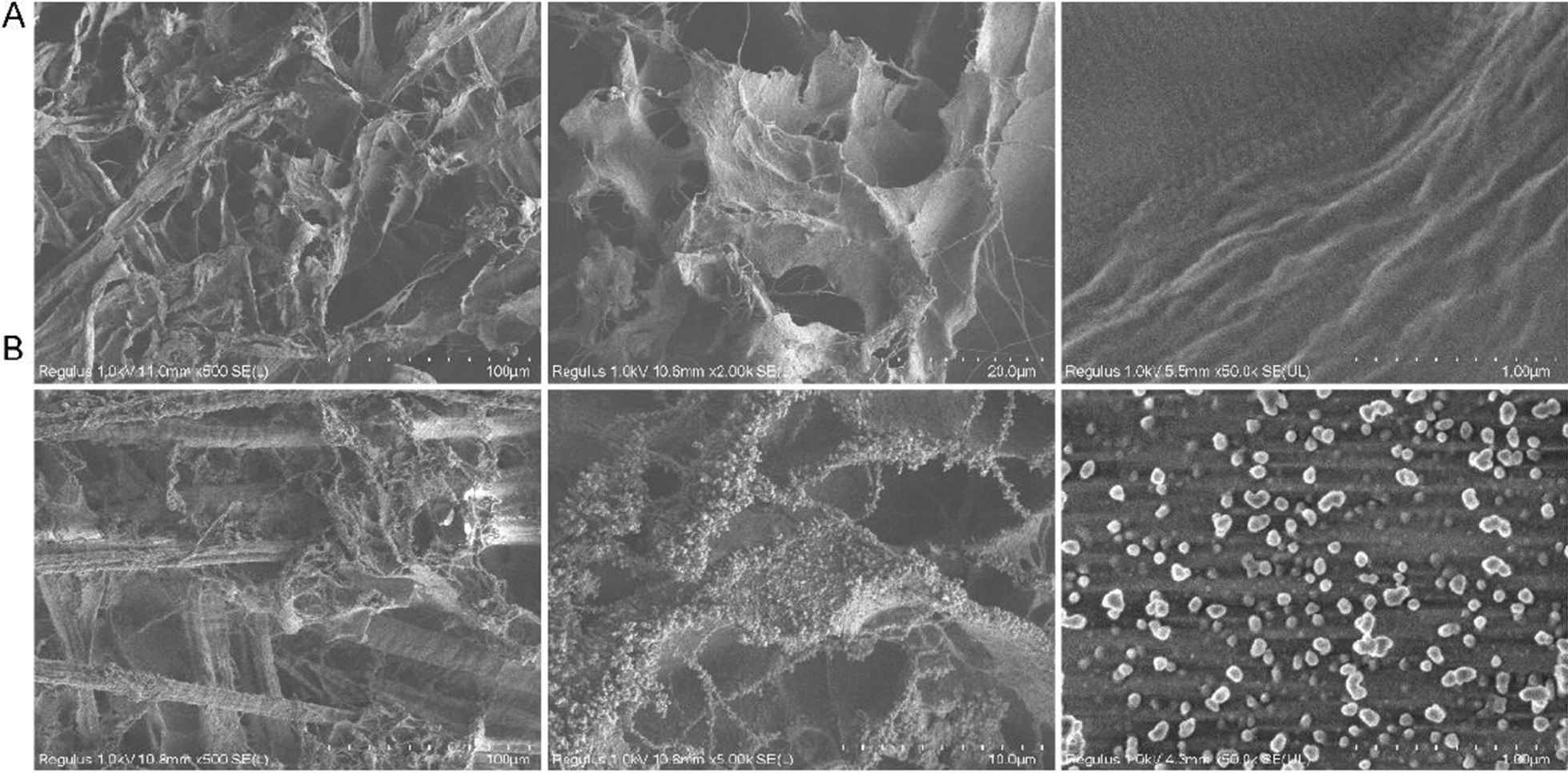
Scanning electron microscopy of collagen sponges and collagen sponges coated with DFSCs-sEVs. **A** Scanning electron microscopy of collagen sponges and the surface of the collagen sponge was porous. **B** Scanning electron microscopy of collagen sponges coated with DFSCs-sEVs, which confirmed the presence of the DFSCs-sEVs particles

To evaluate the effects of DFSCs-sEVs on periodontal regeneration in vivo, we utilized a collagen sponge as a carrier to deliver DFSCs-sEVs to the defective periodontal area in rats. SEM images showed that the surface of the collagen sponge was porous (Fig. 6A) and turned coarse and gritty when loaded with DFSCs-sEVs (Fig. 6B).

**Fig. 7:**
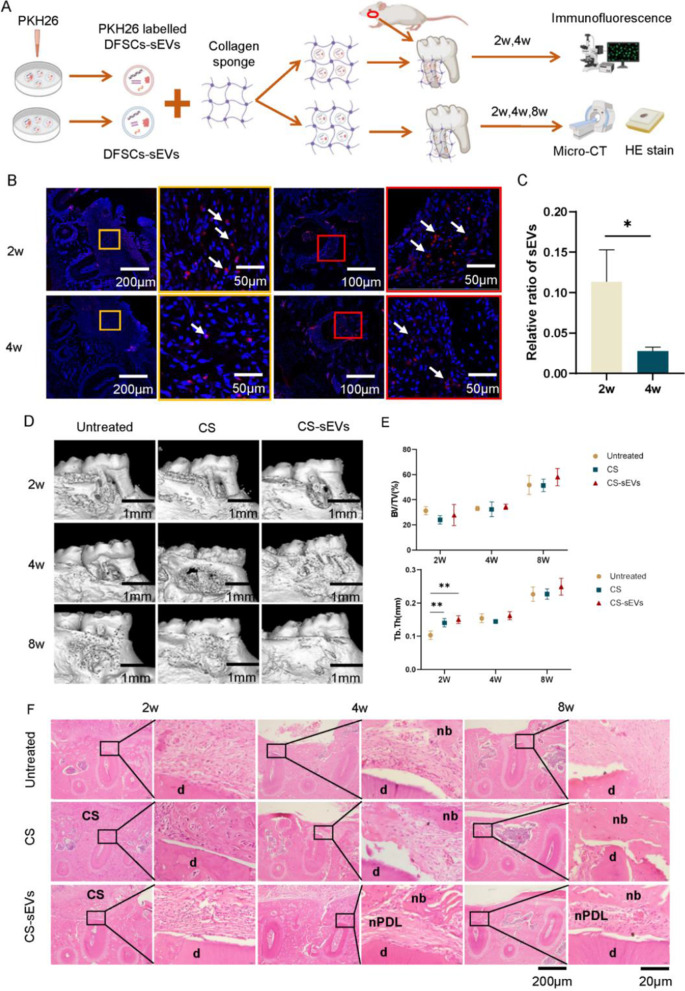
DFSCs-sEVs promoted periodontal tissue regeneration in rats. **A** In vivo experiment design. **B** In vivo location of DFSCs-sEVs. Cell nuclei were stained with DAPI (blue), and DFSCs-sEVs were labelled with PKH26 (red). The white arrows show the DFSCs-sEVs. **C** Quantification of DFSCs-sEVs. **D** Representative micro-CT reconstruction images showing new bone formation in different groups at 2–8 weeks. **E** Quantitative micro-CT assessment of BV/TV (%), Tb.Th (mm). **F** Histological evaluation of periodontal regeneration. CS, collagen sponges; nb, new bone; nPDL, new periodontal ligament; d: dentin. *p < 0.05; **p < 0.01

The results of the micro-CT revealed that few periodontal ligament-like tissues and new bone were formed at 2 weeks after transplantation, while visible newly formed tissue was observed at 4 weeks after transplantation in three groups. After 4 weeks of transplantation, scattered new bone fragments were formed in the collagen sponge group, while diffuse bone formation was observed in the DFSCs-sEVs group, and a denser new bone formation as observed in the DFSCs-sEVs group compared to the other 2 groups (Fig. 7D). Additionally, we found that trabecular thickness in the DFSCs-sEVs group was significantly higher than that in the untreated group at 2 weeks after surgery (Fig. 7E).

Furthermore, periodontal ligament-like tissue between cementum and new bone was observed to different degree in the DFSCs-sEVs group at 2, 4, and 8 weeks after transplantation in all three groups. After the 4 weeks interval, in comparison with the other two groups, the DFSCs-sEVs group showed highly cellular periodontal tissue, with cells perpendicular to the cementum and alveolar bone, presenting the most healing signs, which was consistent with micro-CT results. Connective tissue formation was observed in the untreated and collagen sponge group. Nevertheless, none of the collagen fibrils have stripes that are a distinct feature of the periodontal ligament orderly arranged and embedded between the cementum, covering the root of the tooth and the inner wall of the alveolar bone socket (Fig. 7F).

To explore the localization and survival of DFSCs-sEVs, PKH26-labelled DFSCs-sEVs were transplanted onto the defective periodontal area in rats. After 2 weeks of transplantation, labelled DFSCs-sEVs were observed in the newly formed periodontal ligament and soft tissue of the defect area, while less labelled DFSCs-sEVs were observed after 4 weeks of transplantation (Fig. 7B, C). This indicated that DFSCs-sEVs are involved in the formation of new periodontal ligament-like tissue and new bone in the defective periodontium of rats.

## Discussion

Generation of a functional attachment of periodontal ligament fibres in defective periodontal tissues has been a major challenge in periodontal tissue engineering [1].

Many studies have focused on enhancing periodontal tissue regeneration by applying mesenchymal stromal/stem cells (MSCs) in the treatment strategies. Recently, there has been a paradigm shift in the therapeutic mechanism of MSCs in tissue repair from one based on cellular differentiation and replacement to another based on secretion and paracrine signalling. Adipose-derived stem cells (ADSCs) and their exosomes, stem cells from human exfoliated deciduous teeth (SHED)-derived conditioned exosomes, sEVs from lipopolysaccharide-preconditioned dental follicle cells, and bone marrow mesenchymal stem cell-derived sEVs enhanced periodontal ligament cell functions in vitro and promoted periodontal regeneration in experimental animals [26–31].

In this study, we found that DFSCs-sEVs enhanced the proliferation, migration, and osteogenic differentiation of PDLSCs in vitro. Thereafter, DFSCs-sEVs loaded on a collagen sponge could significantly promote tissue regeneration of the defective periodontal tissue in rats. Consequently, DFSCs-sEVs showed a very promising application potential for periodontal tissue engineering.

We isolated sEVs from the supernatant of DFSC culture media using a commercial kit. To avoid contamination with sEVs from FBS, the cells were cultured in a serum-free media for 48 h before the supernatant was harvested. We found that DFSCs-sEVs could be internalized by PDLSCs through the endocytic mechanism. sEVs enhanced proliferation, cell migration, and osteogenesis differentiation of PDLSCs in vitro, which was consistent with the previous reports [27, 29–31]. Such consistency with the former reports may reflect that the feasibility of sEVs to a larger extent; however, the fluctuation of the cell concentration should be considered. For instance, we found the capability of 50 µg/mL DFSCs-sEVs in promoting the proliferation and migration of PDLSCs was not as evident as that of 10 µg/mL in our study, while 50 µg/mL had been recommended as the optimal concentration in previous researches on ADSC-sEVs, which promoted the proliferation of fibroblasts better than 100 μg/mL ADSC-sEVs [32]. Nevertheless, 100 µg/mL DFSCs-sEVs could be used as a treatment method for experimental periodontitis in rats and are efficient in the promotion of the periodontal ligament cell proliferation extracted from periodontitis patients [29]. Considering that the uptake of sEVs by target cells is normally viewed as a process of saturation [18, 33], we selected the 10 μg/mL concentration for subsequent experiments. The discrepancy in the dosage of sEVs that affect target cells might be certainly due to the phenotypic difference of the origin cells, as well as the local microenvironment after transplantation, such as the inflammatory microenvironment [11], which significantly differed from the ‘cargo’ content of sEVs secreted under normal circumstances [34].

In addition to the proliferation and migration, osteogenic differentiation of PDLSCs is also very important for periodontal tissue regeneration. We demonstrated that DFSCs-sEVs could promote the osteogenic ability of PDLSCs, with the expression levels of osteogenic related proteins and genes (RUNX2 [35], BSP [36]) upregulated in the early stage of osteogenesis but are not obvious in the later stage of osteogenesis. By comparison, the raising expression level of COL1 might be related to the matrix deposition. We hypothesize that this result could be attributed to the synergistic participation of PDLSCs in the formation of periodontal ligament and bone in the process of periodontal tissue regeneration [37–39], as revealed in our in vivo experiment.

Previous studies have indicated that sEVs could activate Wnt, BMP, AKT, and Mitogen-activated protein kinases (MAPK) signalling pathways, which consequently resulted in the promotion of tissue repairment and regeneration [25, 28, 40, 41]. Likewise, our transcriptome sequencing results showed that MAPK signalling pathways were involved in DFSCs-sEVs-mediated PDLSCs proliferation. MAPK, belonging to a large family of serine-threonine kinases, forms the major cell-proliferation signalling pathway from the cell surface to the nucleus [42–44]. MAPK signalling pathways mainly include the extracellular signal-regulated kinase 1/2 (ERK1/2), c-Jun N-terminal kinase (JNK), p38 mitogen-activated protein kinase (p38 MAPK), and ERK5. In this study, we observed that DFSCs-sEVs rapidly activated p38 MAPK and ERK1/2 signalling pathways; however, the DFSCs-sEVs-promoted proliferation of PDLSCs was not affected upon inhibition of the ERK1/2 signalling pathway. This implies that the ERK1/2 signalling pathway may not be the main pathway through which DFSCs-sEVs regulate the proliferation of PDLSCs. A previous study showed that MSCs-sEVs promoted the proliferation and migration of periodontal ligament cells by activating the ERK signalling pathway [27]. Such differences may arise due to the different donor and sources of sEVs, which generated different sEVs ‘cargoes’ as previously speculated [14, 45–47]. Several protein or cell factors have been proven to induce proliferation of cells via the p38 MAPK pathway [48–50]. We also found that the p38 MAPK signalling pathway played a critical role in DFSCs-sEVs-mediated PDLSCs proliferation. However, there may be other mechanisms for regulating the proliferation of PDLSCs because inhibition of the p38MAPK signalling pathway did not virtually impair the proliferation of PDLSCs.

To explicitly observe sEVs, many scholars have labelled sEVs with membrane affinity dyes such as PKH26 and DiO [21]. In our study, at 2- and 4-weeks post transplantation, PKH26-labelled DFSCs-sEVs were observed in the defected area. Moreover, the number of DFSCs-sEVs labelled with PKH26 retained in the defected area decreased significantly at 4 weeks.

DFSCs-sEVs could be rapidly internalized by PDLSCs in vitro, subsequently activating the relevant pathways and regulating the proliferation, migration, and osteogenic differentiation of PDLSCs. We therefore hypothesized that DFSCs-sEVs transplanted to the defect site might be internalized by nearby cells and then participate in the regulation of a series of biological behaviours of the surrounding target cells. This can be supported by the observed phenomenon that a wider range of new bone and ordered periodontal ligament-like complexes were inserted between the new bone and cementum in the DFSCs-sEVs group rats.

Overall, this study provided a theoretical foundation for "cell-free" therapy in periodontal tissue engineering. Compared with traditional cell therapy, cell-free therapy based on sEVs offers a multitude of advantages, including profuse sources, stable structure, convenient storage, and easy modification of loaded cargo [51, 52]. Although DFSCs-sEVs have already been proven to regenerate damaged periodontal tissues in rats successfully, the effective remedy in humans remains to be explored pragmatically and clinically. The current experimental animal model for periodontal tissue regeneration cannot comprehensively reflect the chronic inflammatory process of periodontitis in clinical patients. Further studies are needed to explore the efficacy of cell-free therapy in periodontitis patients.

## Conclusion

**Fig. 8:**
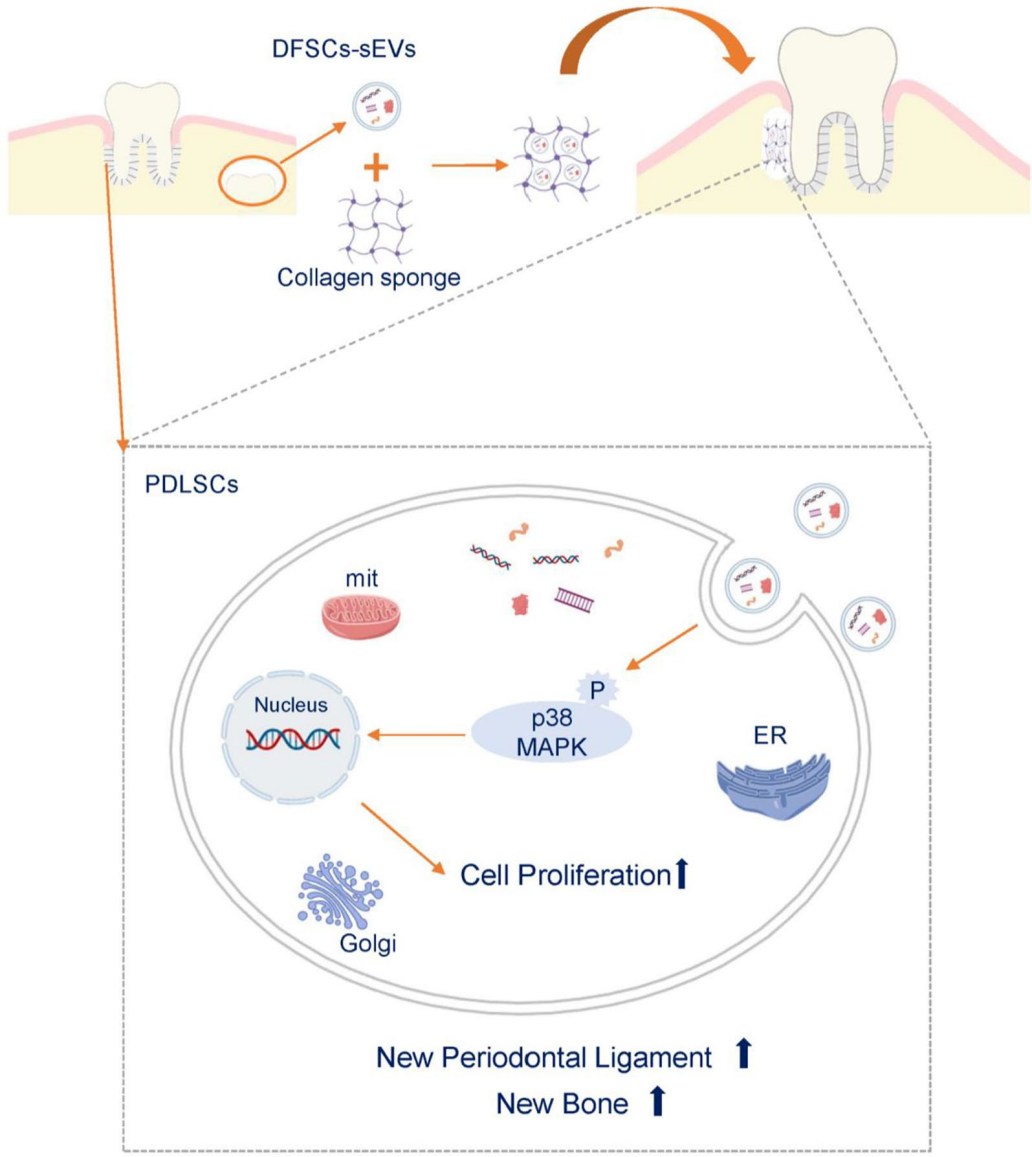
A proposed underlying mechanism of DFSCs-sEVs-mediated promotion of periodontal tissue repair and regeneration. DFSCs-sEVs promoted the formation of new periodontal ligament-like structures and new bone in the defective periodontal tissues in rats by promoting the proliferation of PDLSCs through activation of the p38 MAPK signalling pathway

In this study, DFSCs-sEVs promoted the formation of new periodontal ligament-like structures and new bone in the defective periodontal tissues in rats by promoting the proliferation, migration, and osteogenic differentiation of PDLSCs; these physiological processes may be partially attributed to the activation of the p38 MAPK signalling pathway (Fig. 8). Our findings provide an experimental basis for the development of cell-free therapeutic strategies in periodontal tissue regeneration.

## Supplementary Information

**Additional file 1.** Supplementary Results and Full-length gel images of western blot.

## Data Availability

Not applicable.

## Abbreviations

ADSC: Adipose stem cell
ALP: Alkaline phosphatase
BSP: Bone sialoprotein
BV: Bone volume
CDKN1C: Cyclin-dependent kinase inhibitor 1C
COL1: Collagen 1
CRISPLD2: Cysteine-rich secretory protein LCCL domain-containing 2
CS: Collagen sponges
DFSCs: Dental follicle stem cells
DMSO: Dimethyl sulfoxide
EGR-3: Early growth response-3
ERK: Extracellular signal-regulated kinase
FGF7: Fibroblast growth factor 7
GAPDH: Glyceraldehyde 3-phosphate dehydrogenase
GO: Gene ontology
HSP90: Heat shock protein 90
JNK: C-Jun N-terminal kinase
KEGG: Kyoto Encyclopedia of Genes and Genomes
MAPK: Mitogen-activated protein kinases
MCP-1: Monocyte chemoattractant protein 1
micro-CT: Micro-computed tomography
MSC: Mesenchymal stem cell
NTA: Nanoparticle tracking analysis
PDLSCs: Periodontal ligament stem cells
PVDF: Polyvinylidene difluoride
RUNX2: Runt-related transcription factor 2
SD: Sprague–Dawley
sEVs: Small extracellular vesicles
SHED: Stem cells from human exfoliated deciduous teeth
SLC20A1: Solute carrier family 20 member 1
SOCS1: Suppressor of cytokine signalling 1
SOD2: Superoxide dismutase 2
Tb.Th: Trabecular thickness
TRIB3: Tribbles pseudokinase 3
TSG101: Tumour susceptibility 101
TV: Tissue volume
