# Supplementary material for: Small extracellular vesicles from dental follicle stem cells provide biochemical cues for periodontal tissue regeneration
Source: Stem Cell Res Ther. 2022 Mar 3;13:92. doi: 10.1186/s13287-022-02767-6 (PMC8895915; doi:10.1186/s13287-022-02767-6)

**Small extracellular vesicles from dental follicle stem cells provide biochemical cues for periodontal tissue regeneration**

Liya Ma^1,2†^

Affiliations: Yunnan Key Laboratory of Stomatology & Department of Dental Research, The Affiliated Stomatology Hospital of Kunming Medical University, Kunming, Yunnan, 650500, PR China

Phone number: +8613354658912

Email [maliya1009@163.com](mailto:maliya1009@163.com)

Nangquan Rao^1†^

Affiliations: Yunnan Key Laboratory of Stomatology & Department of Dental Research, The Affiliated Stomatology Hospital of Kunming Medical University, Kunming, Yunnan, 650500, PR China

Hui Jiang^1^

Affiliations: Yunnan Key Laboratory of Stomatology & Department of Dental Research, The Affiliated Stomatology Hospital of Kunming Medical University, Kunming, Yunnan, 650500, PR China

Yuzhe Dai^1^

Affiliations: Yunnan Key Laboratory of Stomatology & Department of Dental Research, The Affiliated Stomatology Hospital of Kunming Medical University, Kunming, Yunnan, 650500, PR China

Songtao Yang^1^

Affiliations: Yunnan Key Laboratory of Stomatology & Department of Dental Research, The Affiliated Stomatology Hospital of Kunming Medical University, Kunming, Yunnan, 650500, PR China

Hefeng Yang^1*^

Affiliations: Yunnan Key Laboratory of Stomatology & Department of Dental Research, The Affiliated Stomatology Hospital of Kunming Medical University, Kunming, Yunnan, 650500, PR China

Phone number: +8613708428343

Email [yanghefeng2008@163.com](mailto:yanghefeng2008@163.com)

Jiangtian Hu^2*^

Affiliations: Department of Orthodontics, The Affiliated Stomatology Hospital of Kunming Medical University, Kunming, Yunnan, 650500, PR China

Phone number: +8613769118403

Email [1041818680@qq.com](mailto:1041818680@qq.com)

^*^ Corresponding author.

^†^ These authors contributed equally to this work.


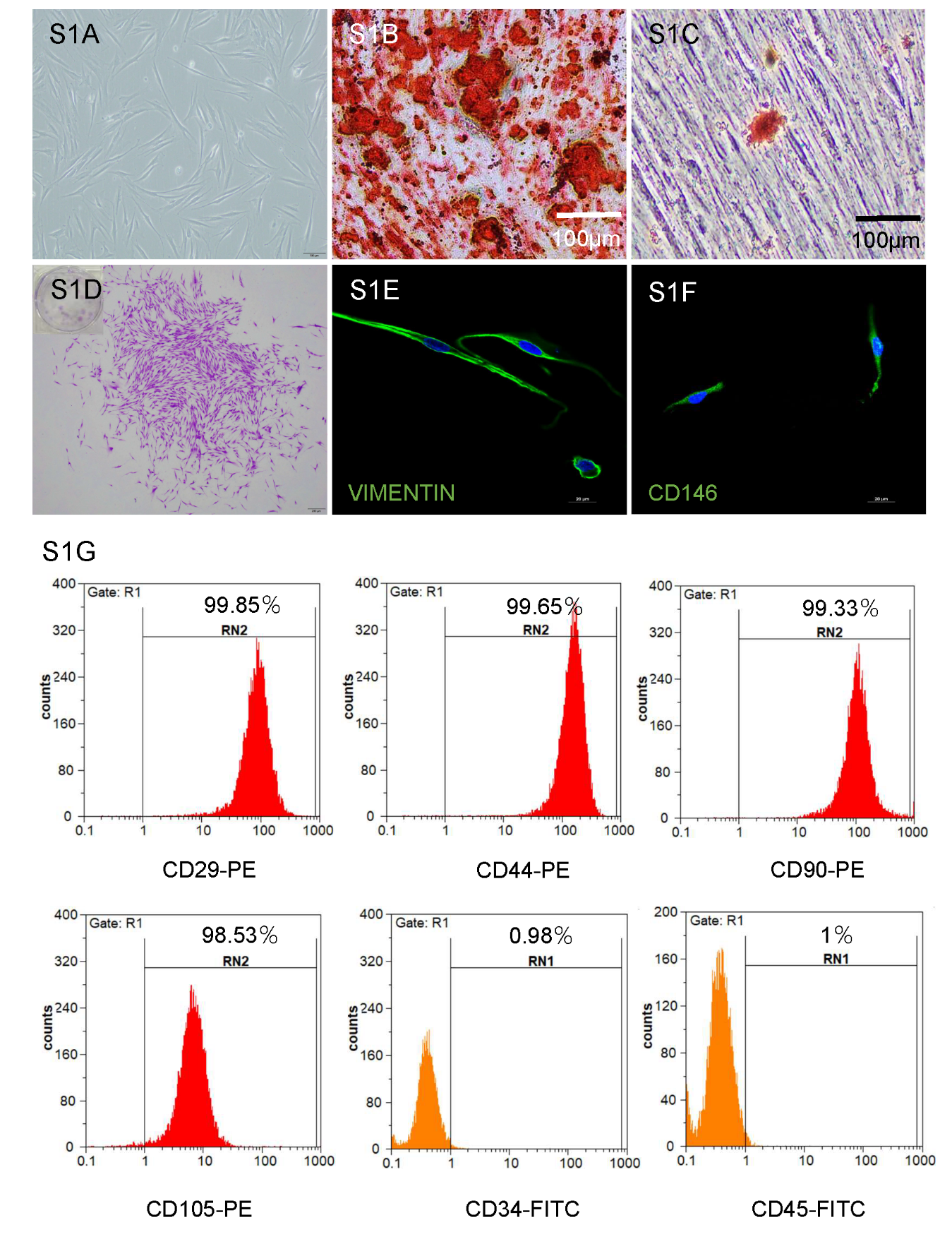


Figure S1. Characteristics of PDLSCs. (A) PDLSCs at passage 3. (B) Osteogenesis. (C) Adipogenesis. (D) Colony formation of PDLSCs. (E) Vimentin expression in passage 3 PDLSCs. (F) CD146 expression in passage 3 PDLSCs. (G) Flow cytometric analysis of surface markers in PDLSCs.


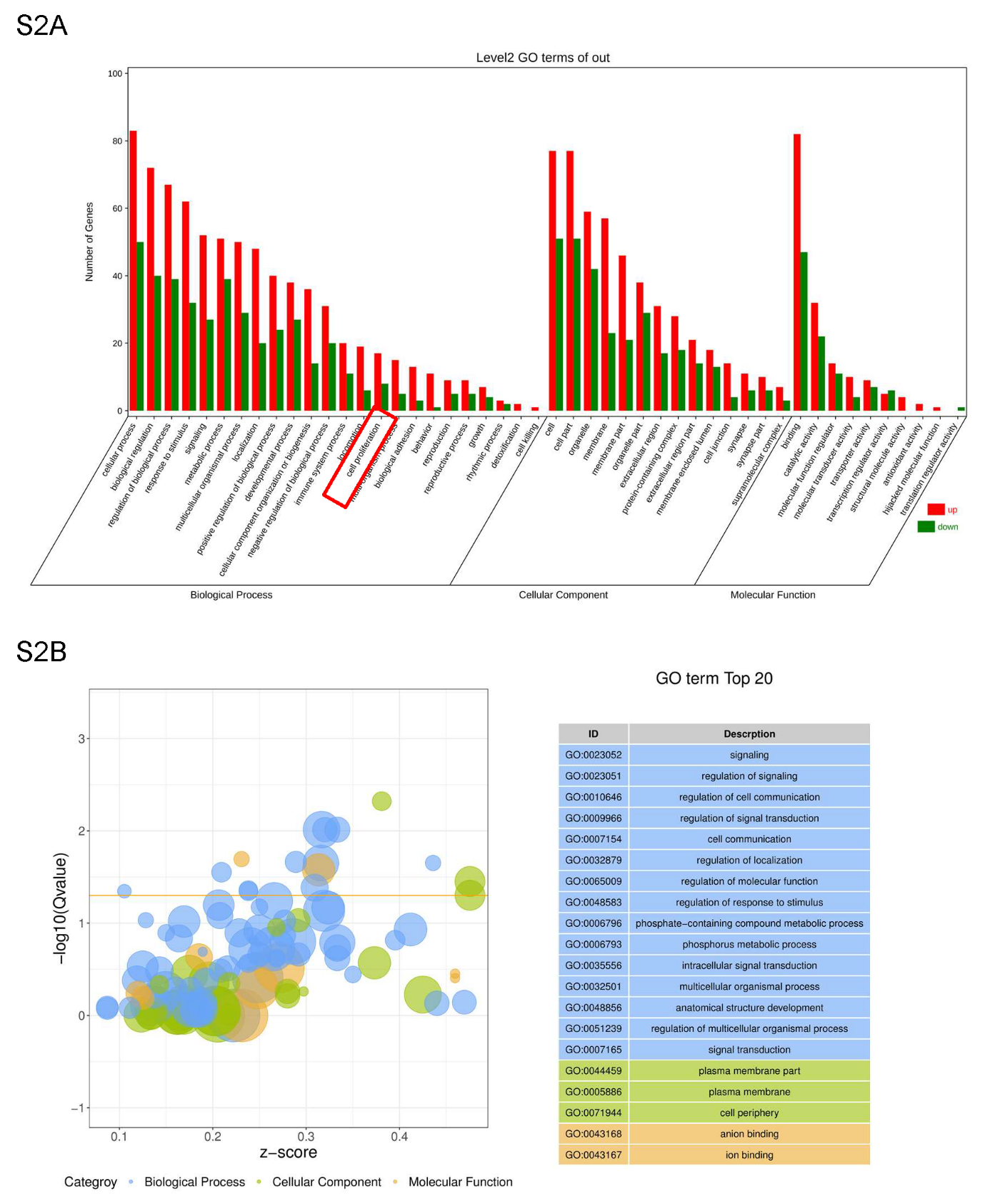
Figure S2. Gene Ontology (GO) function analysis. (A) GO classification bar plot for differently expressed genes in DFSCs-sEVs treated and untreated PDLSCs. (B) GO classification bubble.

Full-length gel images of western blot

Figure 1K


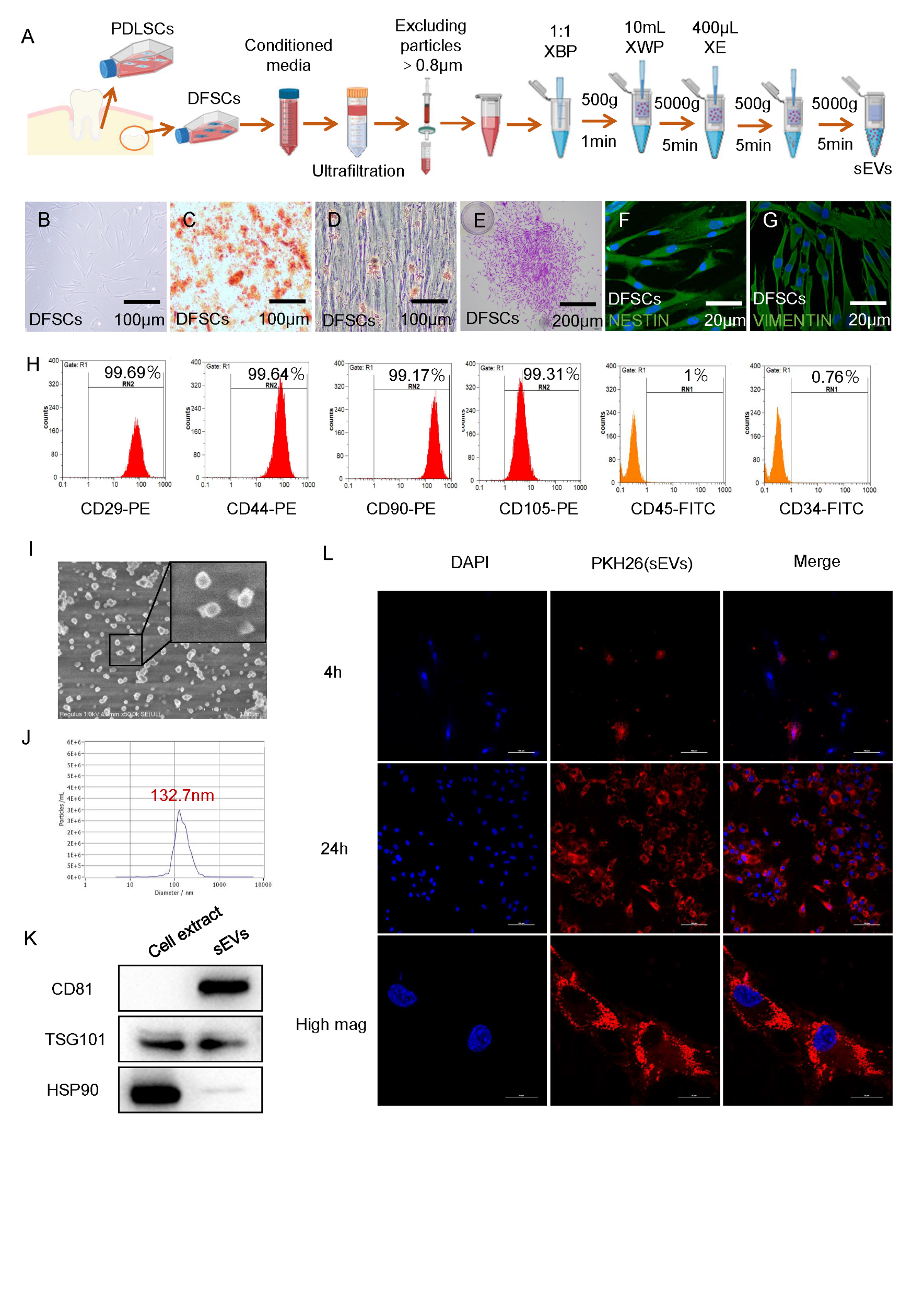


The original blot


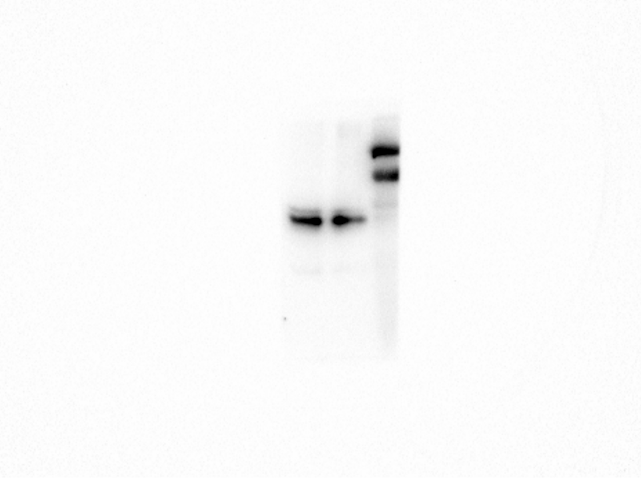
 CD81 26KD TSG101 44KD
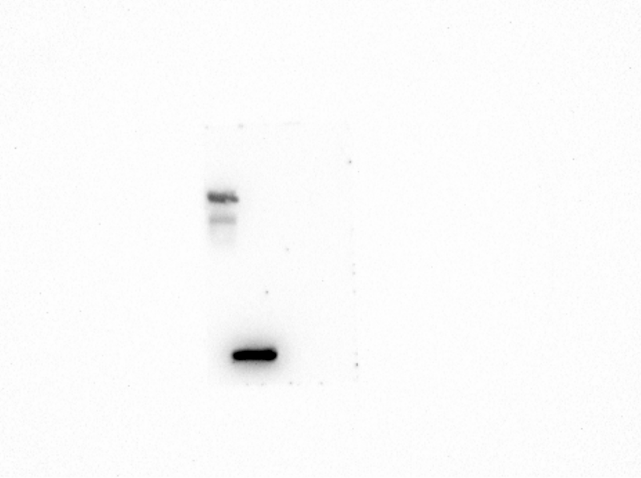


Cell extract

sEVs

sEVs

Cell extract

HSP90 83.3KD


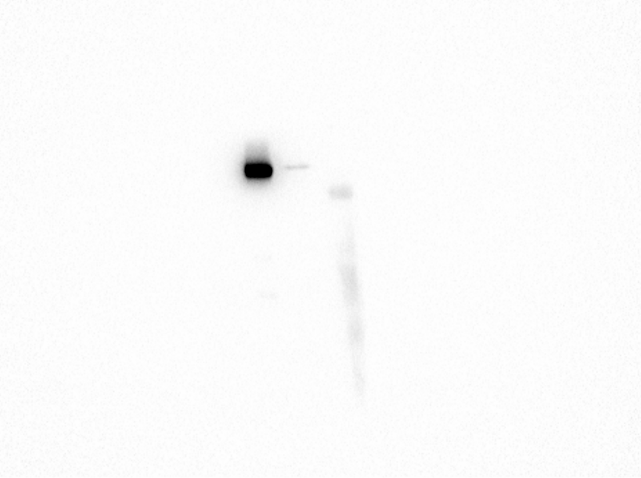


Cell extract

sEVs

Figure 3D


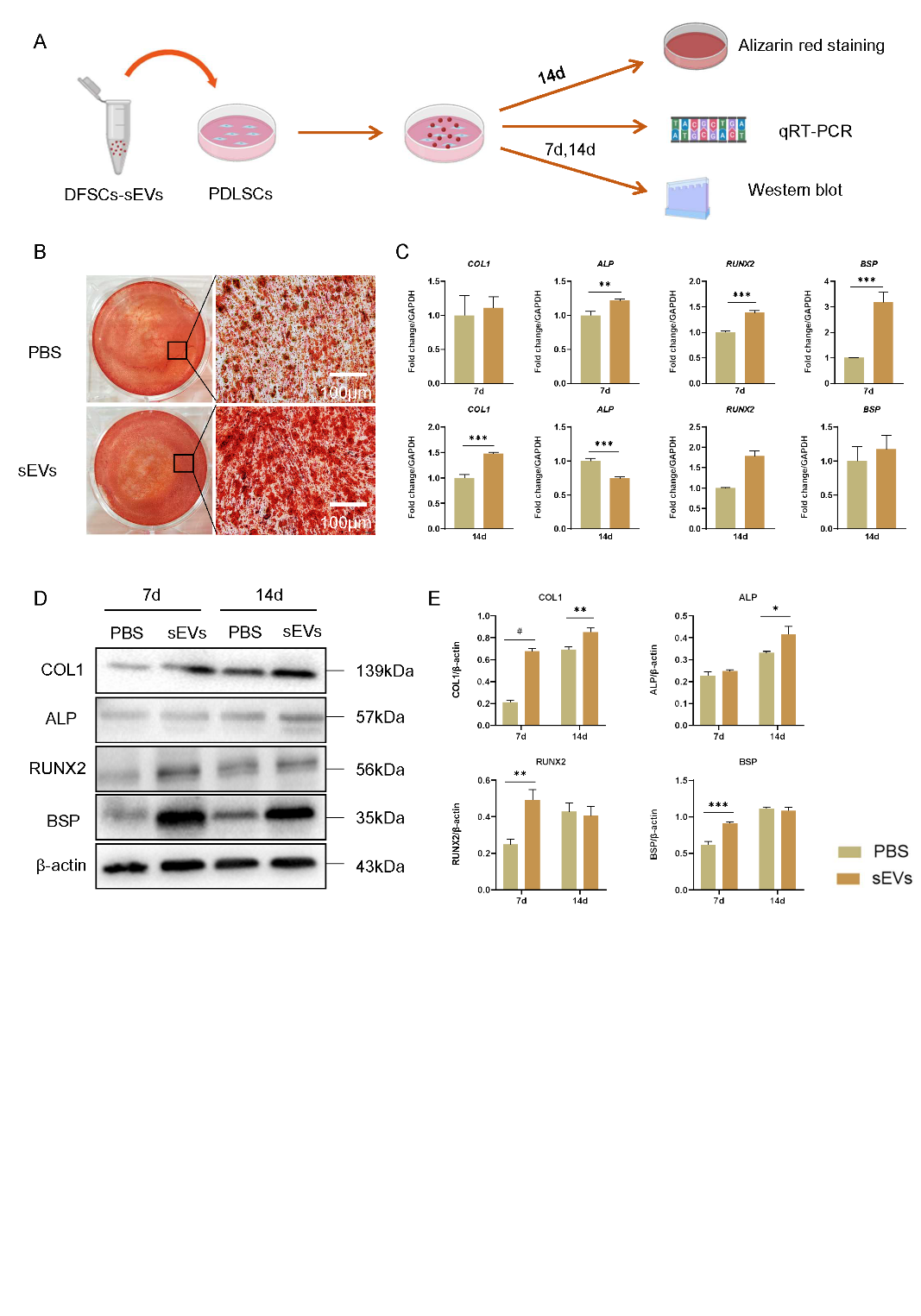


The original blot

COL1 139KD ALP 57KD


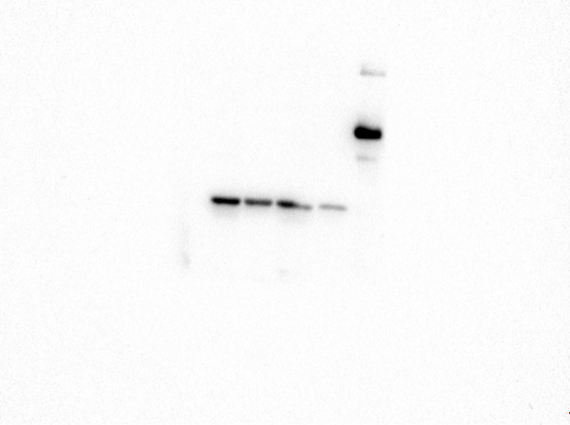

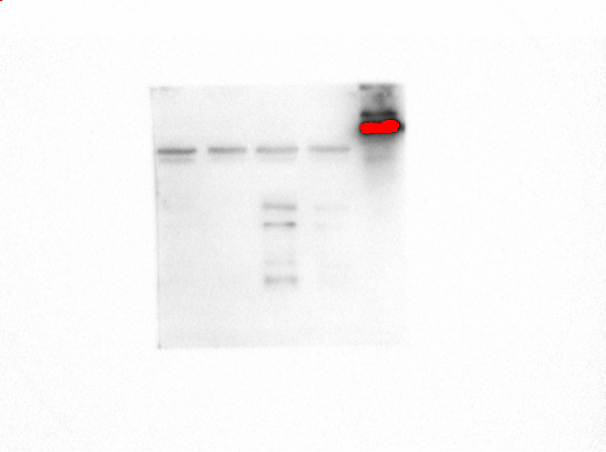


Runx2 56KD BSP 35KD


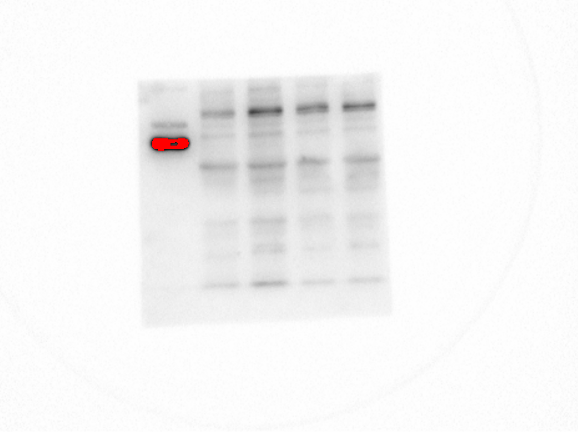

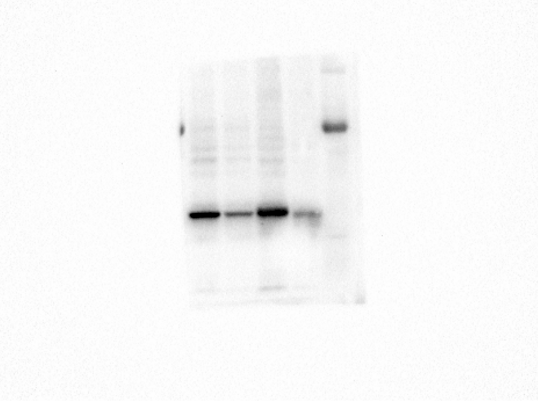


β-actin 43KD


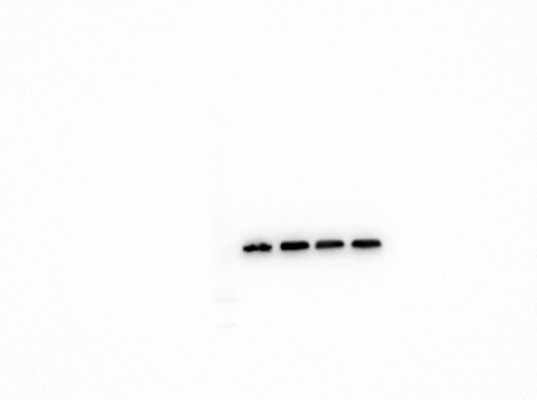


Figure 5B


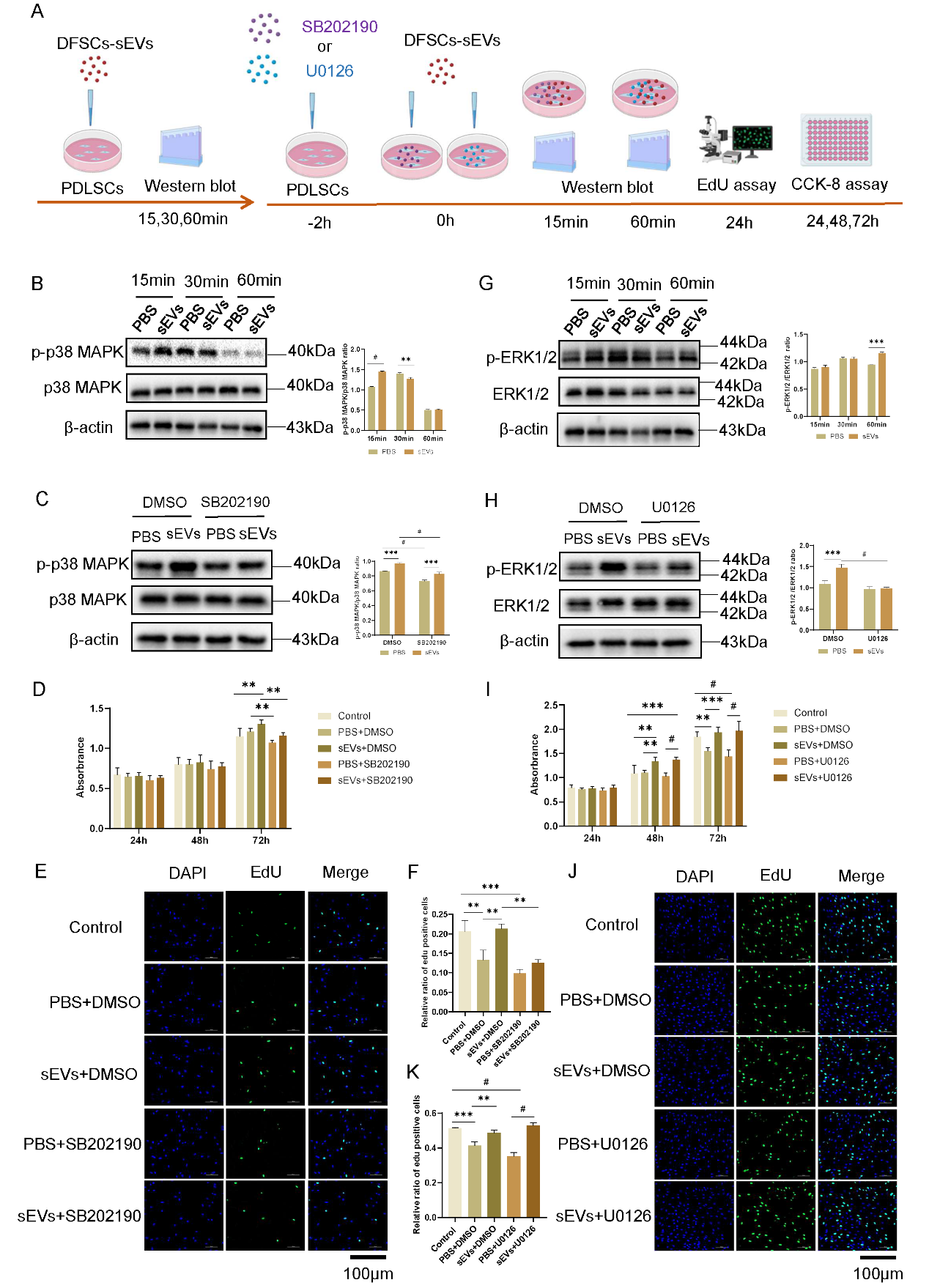


The original blot

p38 40KD p-p38 40KD


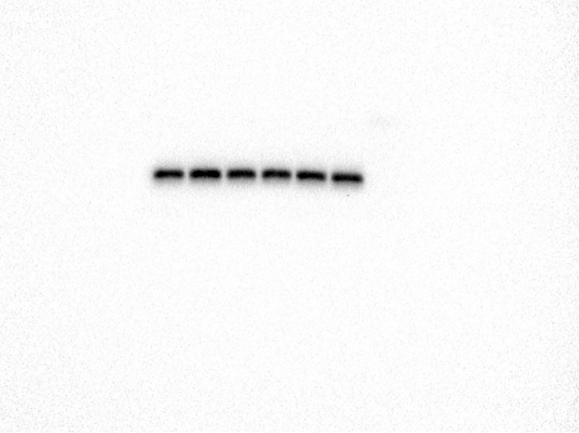

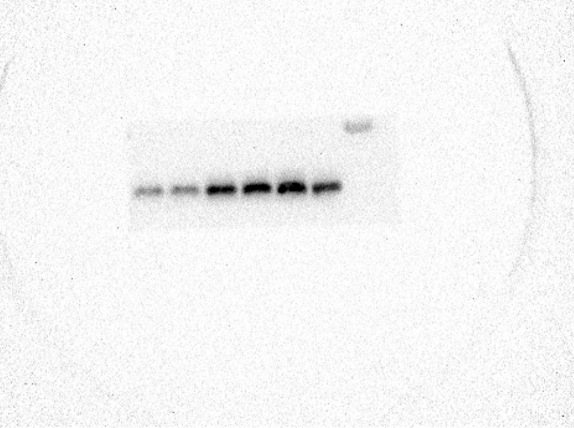


β-actin 43KD


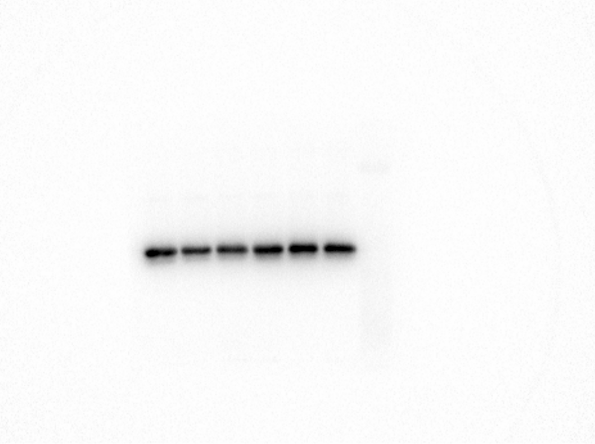


Figure 5C


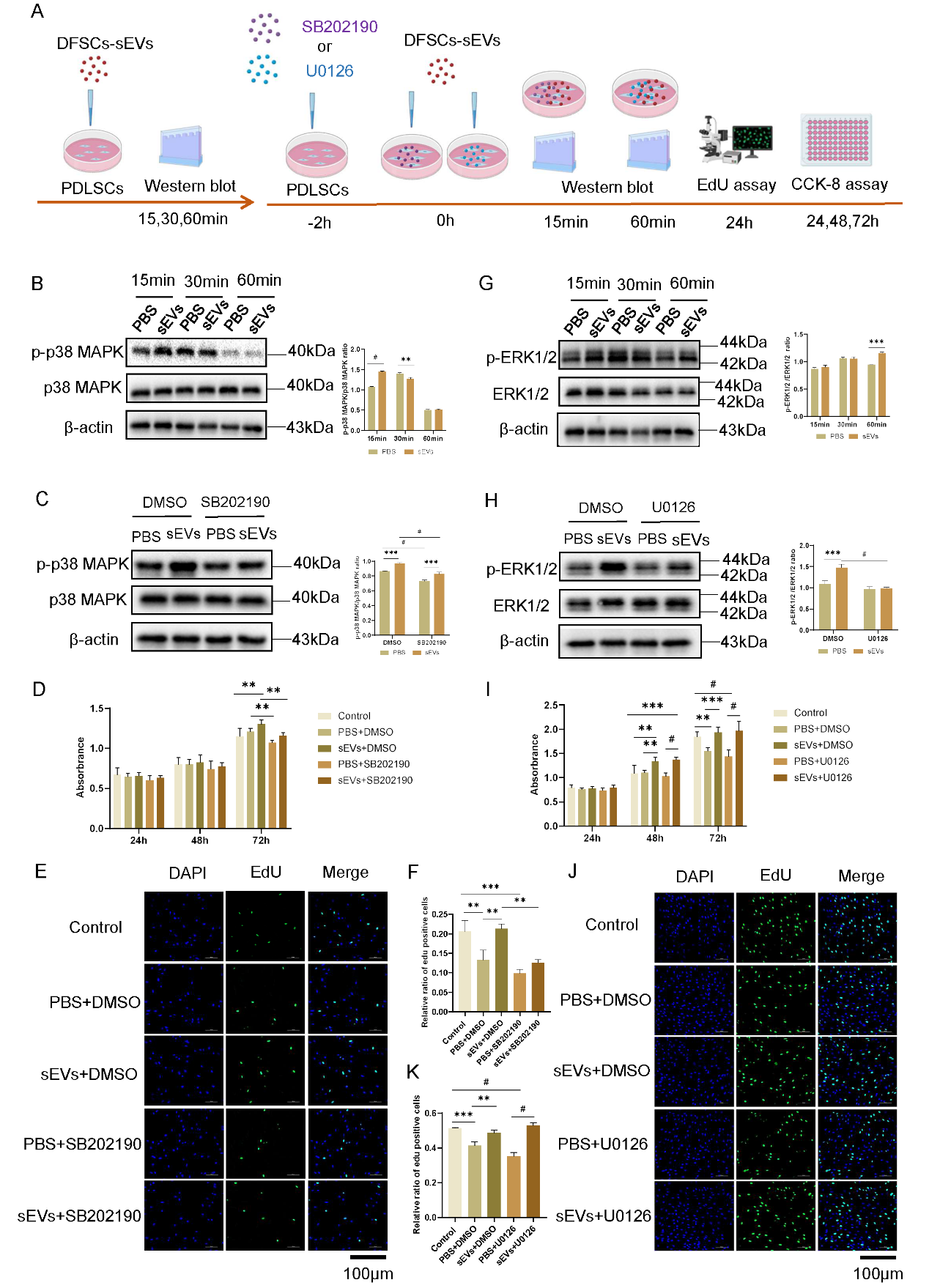


The original blot

p-p38 40KD p38 40KD


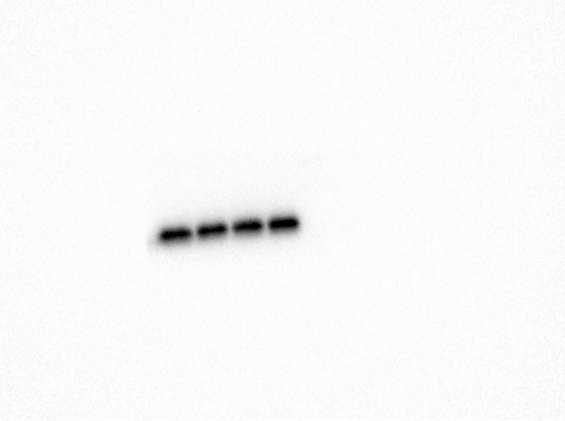

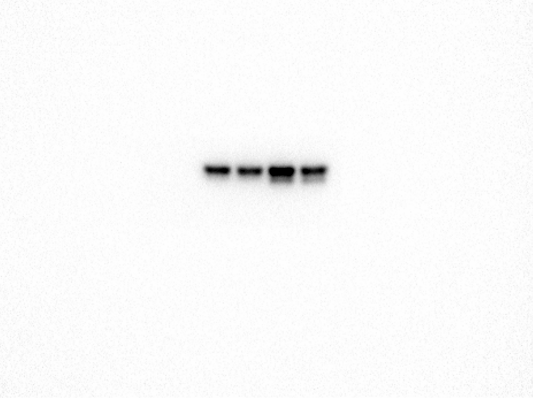


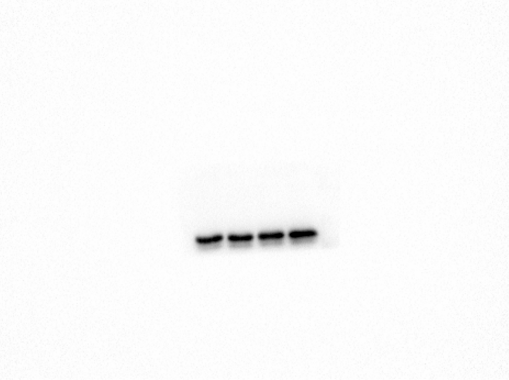
 β-actin 43KD

Figure 5G


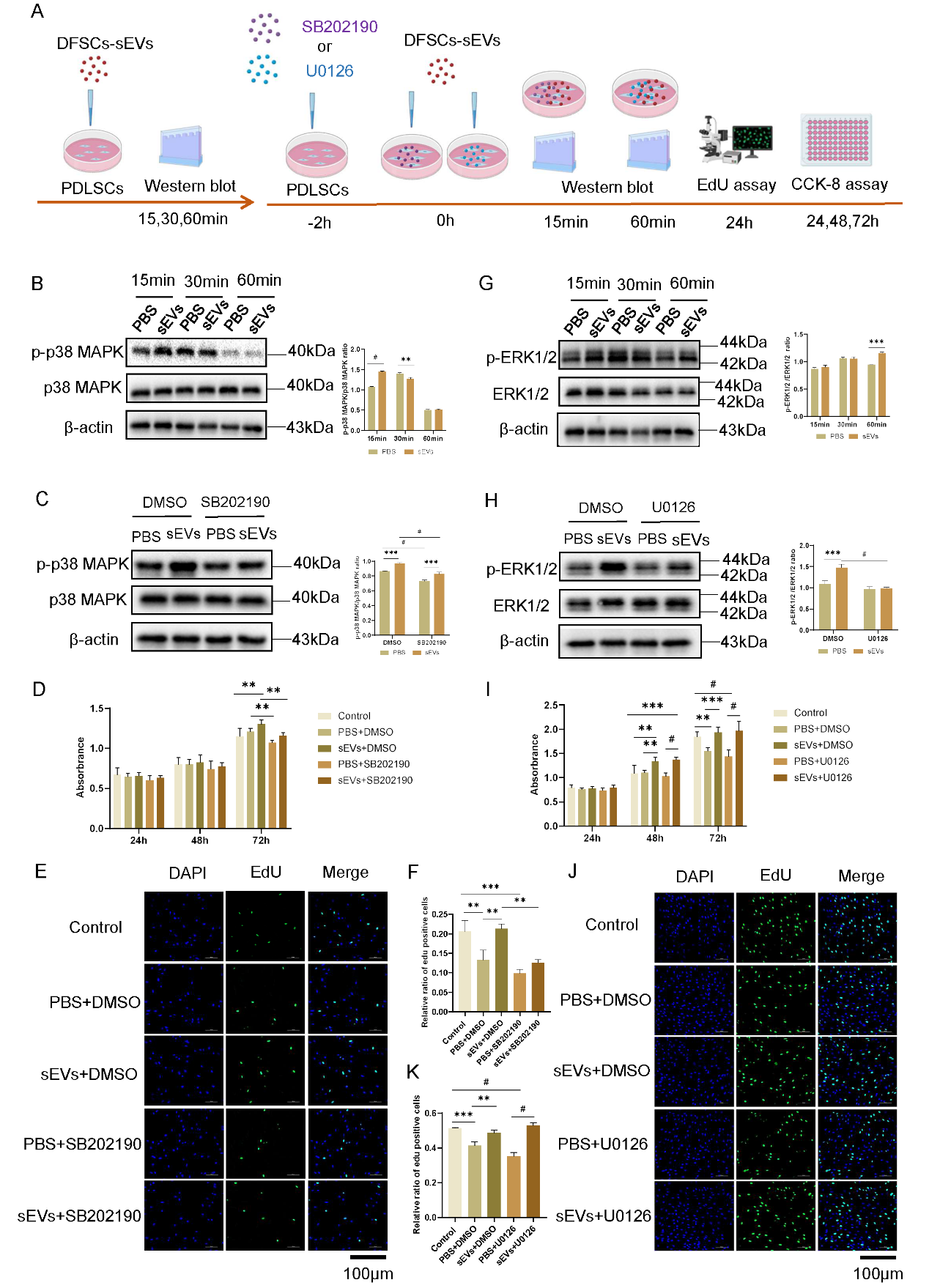


The original blot


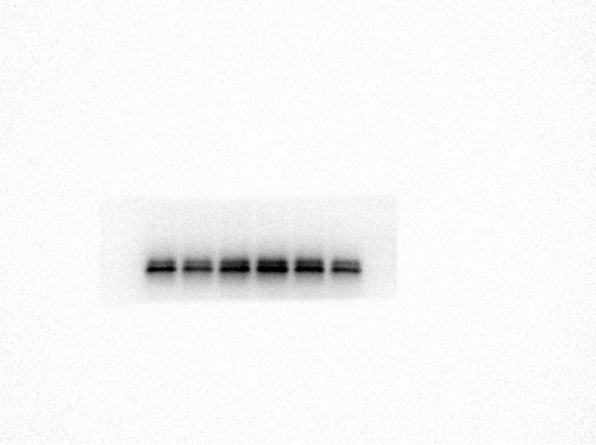
p-ERK1/2 44/42KD ERK1/2 44/42KD


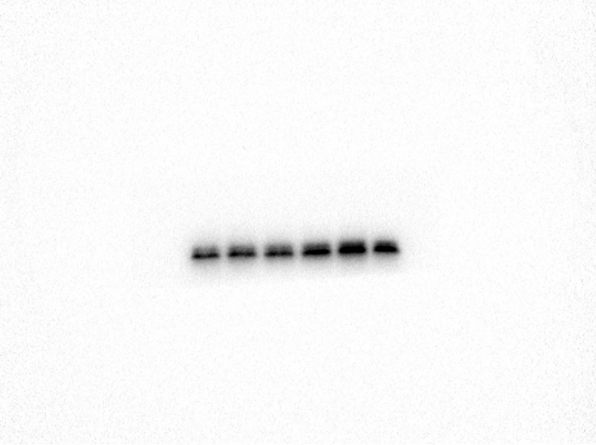


β-actin 43KD


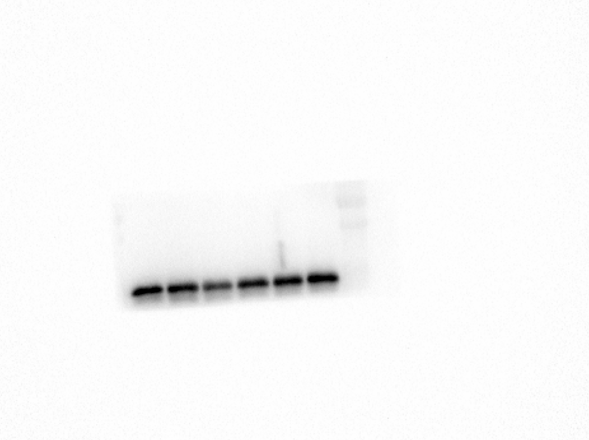


Figure 5H


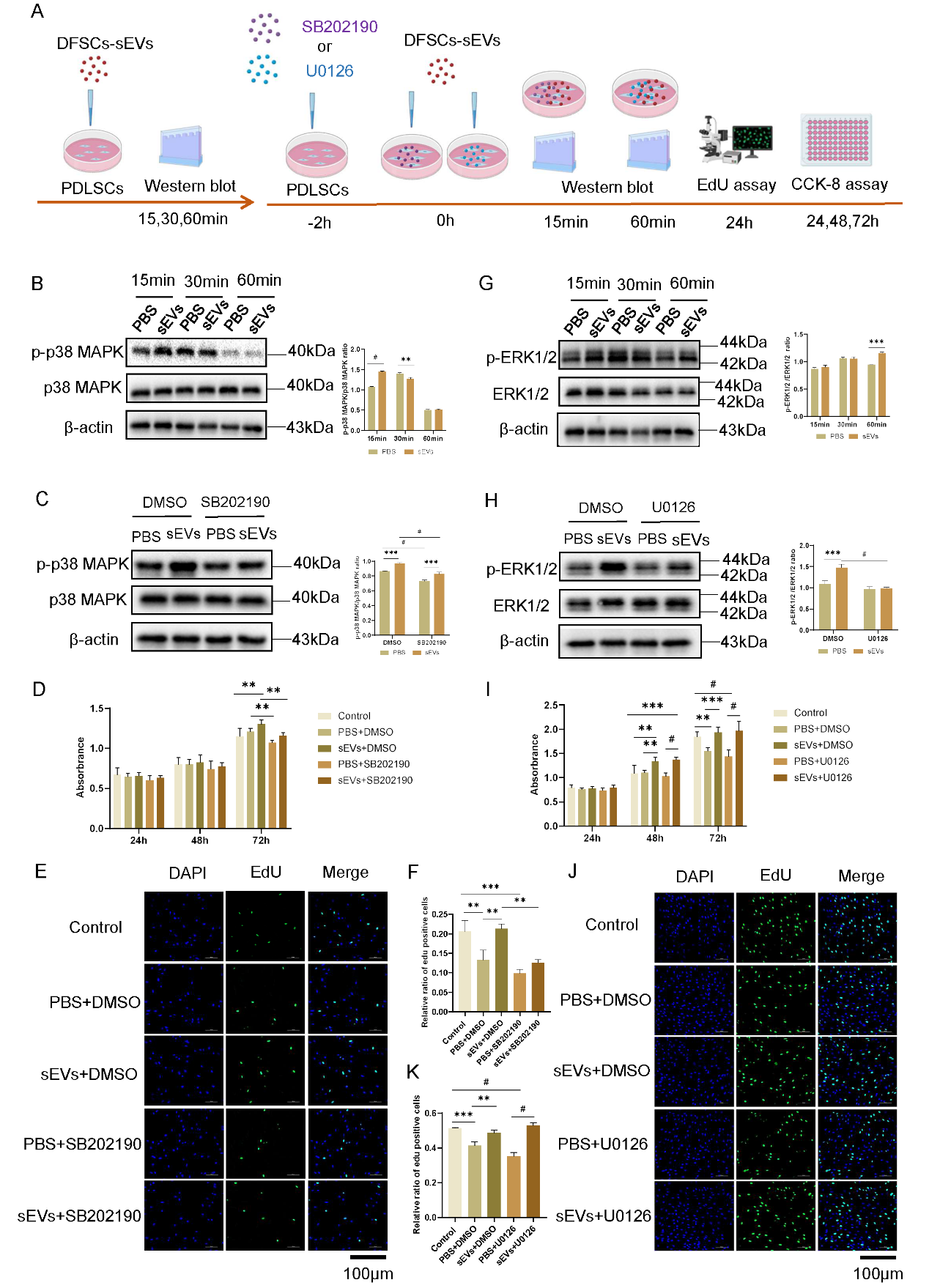


The original blot

p-ERK1/2 44/42KD ERK1/2 44/42KD


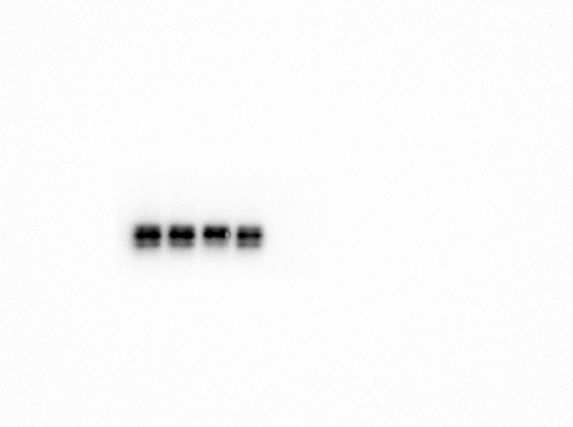

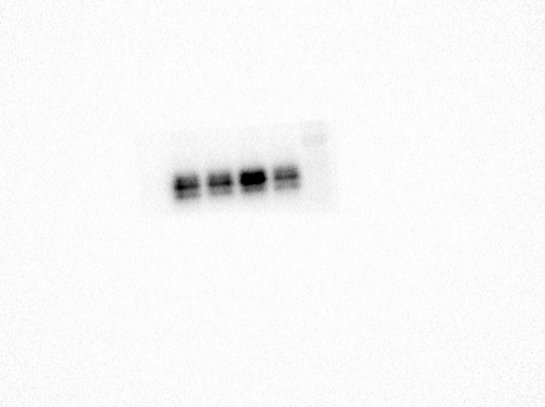


β-actin 43KD


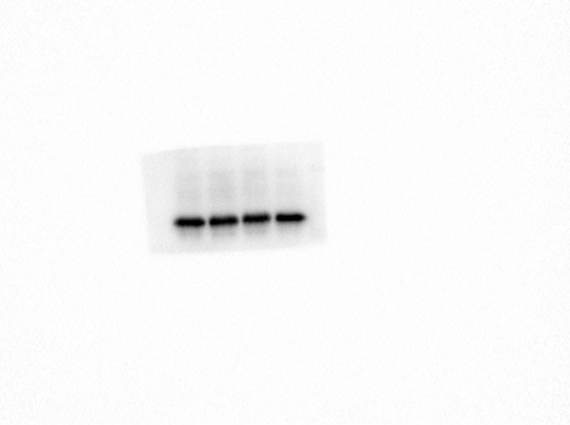

Supplement: Supplementary file 1 — Additional file 1. Supplementary Results and Full-length gel images of western blot. [file 13287_2022_2767_MOESM1_ESM.docx]
